# Periostin blockade overcomes chemoresistance via restricting the expansion of mesenchymal tumor subpopulations in breast cancer

**DOI:** 10.1038/s41598-018-22340-7

**Published:** 2018-03-05

**Authors:** Youya Nakazawa, Yoshiaki Taniyama, Fumihiro Sanada, Ryuichi Morishita, Shoji Nakamori, Koji Morimoto, Kay T. Yeung, Jing Yang

**Affiliations:** 1 Department of Pharmacology, Moores Cancer Center, University of California, San Diego, La Jolla, California USA; 2 Department of Clinical Gene Therapy and Geriatric and General Medicine, Osaka University Graduate School of Medicine, Osaka, Japan; 3 Department of Hepato-Biliary-Pancreatic Surgery, Osaka National Hospital, National Hospital Organization, Osaka, Japan; 4 Osaka Women’s Junior College, Osaka, Japan; 5 Department of Medicine, University of California, San Diego, La Jolla, California USA; 6 Department of Pediatrics, University of California, San Diego, La Jolla, California USA

## Abstract

Recent studies suggest a functional involvement of Epithelial-Mesenchymal Transition (EMT) in tumor chemoresistance. Specifically, EMT is associated with chemoresistance and poor prognosis in triple-negative breast cancer. However, no effective therapy targeting EMT has been developed. Here, we report that periostin, an extracellular matrix protein, was induced upon chemotherapy and tightly correlated with the EMT gene signature and poor prognosis in breast cancer. In triple-negative breast cancer xenografts, chemotherapy upregulated periostin expression in tumor cells, triggered expansion of mesenchymal tumor cells and promoted invasion in residual tumors. Knockdown of periostin inhibited outgrowth and invasion of mesenchymal tumor cells upon chemotherapy. Furthermore, chemotherapy upregulated cancer-specific variants of periostin and application of a blocking antibody specifically targeting those variants overcame chemoresistance and halted disease progression without toxicity. Together, these data indicate that periostin plays a key role in EMT-dependent chemoresistance and is a promising target to overcome chemoresistance in triple-negative breast cancer.

## Introduction

TNBC is an aggressive subtype of breast cancer that is closely related to basal-like breast cancer with a strong EMT gene signature with poor overall prognosis. Due to a lack of estrogen receptor (ER), progesterone receptor (PR) or human epidermal growth factor receptor 2 (HER-2) expressions for targeted therapy in TNBC, its treatment largely consists of cytotoxic chemotherapies with anthracyclines and taxanes. Although a subset of TNBCs are responsive to initial chemotherapy, the probability of early relapse is significantly higher compared to other subtypes of breast cancer^1^. Several studies report that a number of EMT markers are associated with chemoresistance or poor prognosis in TNBCs^2–4^. However, the cause of chemoresistance in patients with TNBC is unclear and there is an urgent need to overcome chemoresistance and prolong therapeutic response.

The EMT program is a critical process during embryo development by which epithelial cells lose epithelial polarity, weaken cell-cell adhesions and obtain a migratory phenotype^5^. Many studies reveal a functional involvement of EMT in various steps of cancer progression: invasion, metastasis, chemoresistance and relapse^6,7^. Therefore, the EMT regulatory pathway has attracted great therapeutic interest in cancer treatment. Specifically, a number of studies showed that tumor cells undergo EMT to escape from chemotherapy and other targeted therapies. However, therapeutic strategy targeting EMT has not been developed to overcome therapy resistance.

Periostin (POSTN, osteoblast-specific factor 2, OSF-2) is a secreted extracellular matrix (ECM) protein, which was originally identified in osteoblasts^8^. Periostin overexpression and function have been implicated in several types of human cancer^9,10^. In this report, we set to determine the functional role of periostin in EMT-mediated chemoresistance in TNBC and to explore its clinical implications in overcoming chemoresistance in TNBC.

## Results

### Chemotherapy upregulates periostin and enriches the mesenchymal population in TNBC xenografts and patient samples

**Figure 1:**
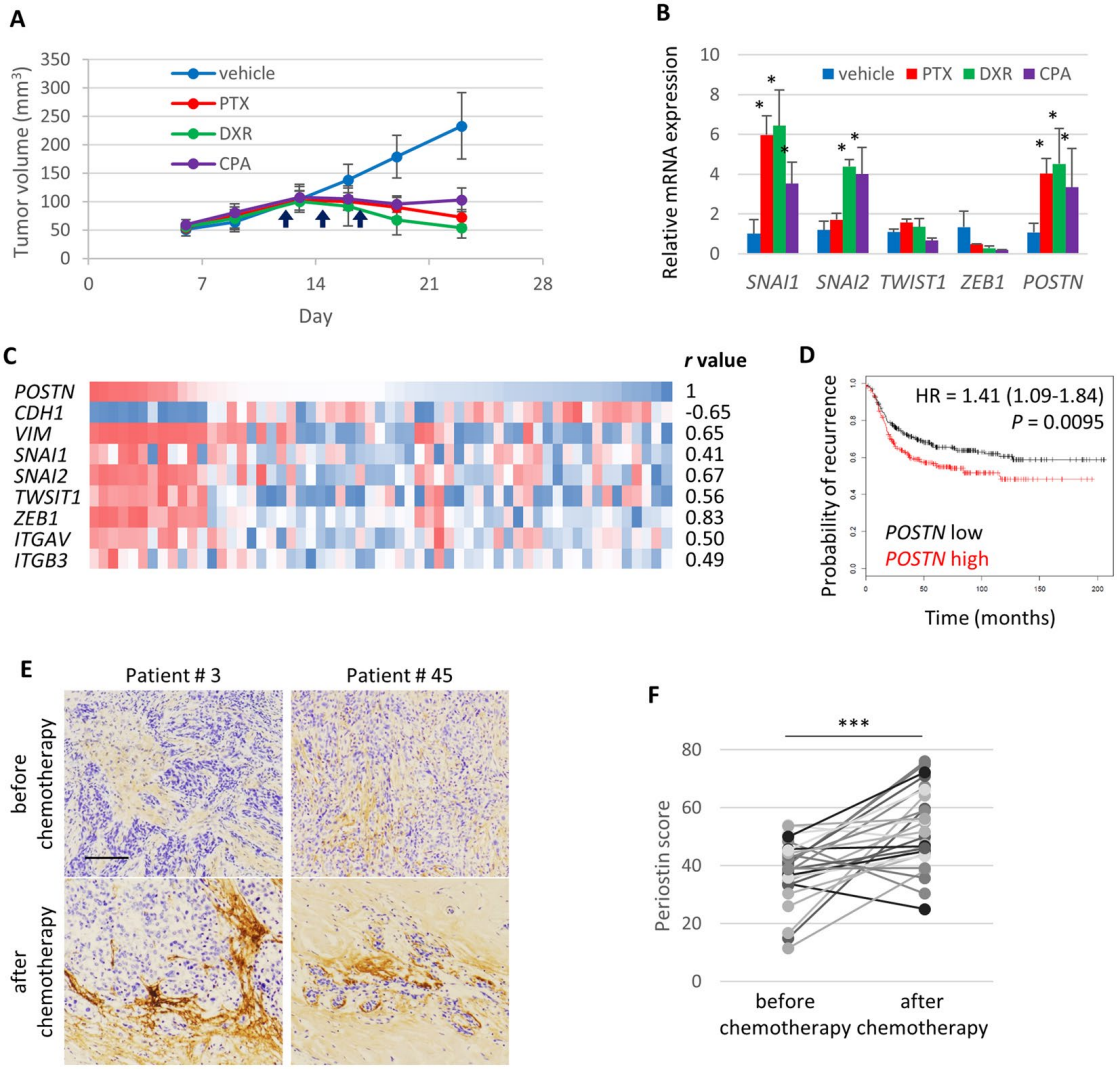
Chemotherapy upregulated periostin expression in chemoresistant triple-negative breast cancer xenografts and human TNBC patient samples. (**A**) Tumor growth curve in response to paclitaxel (PTX), doxorubicin (DXR) and cyclophosphamide (CPA) in MCF10DCIS xenografts. Arrows indicate times of drug administration. Data are presented as mean ± standard error (n = 6). (**B**) Quantitative PCR analysis of indicated genes in tumors harvested at the end of the experiment in **A**. Relative values are presented as mean ± standard error (n = 3). *P < 0.05 versus vehicle-treated control, Student’s *t* test. (**C**) Correlation analysis of *POSTN* expression in 59 human breast cancer cell lines. Heatmap of gene expression and Pearson product-moment correlation coefficient (r value) are shown. (**D**) Patient survival analysis in basal-like breast cancer patients (n = 580) according to *POSTN* expression (low: 66.6%, high: 33.3%). Hazard ratio (HR), 95% confidence intervals. *P* value, log rank test. (**E**) Immunohistochemistry staining of periostin with human TNBC samples. Scale bar, 100 µm. (**F**) Periostin score before and after chemotherapy in 26 matched pair TNBC samples which showed residual disease after chemotherapy. ***P < 0.001, Wilcoxon signed-rank test.

To explore the *in vivo* role of EMT in chemotherapy, we generated tumor xenografts using a human basal-like TNBC model, MCF10DCIS. MCF10DCIS cell line is derived from a premalignant epithelial breast tumor xenograft and lacks the expression of ER, PR, or HER-2. Mice carrying MCF10DCIS human mammary tumors were treated with paclitaxel (tubulin inhibitor, PTX), doxorubicin (DNA intercalater, DXR) and cyclophosphamide (DNA alkylator, CPA), which are standard chemotherapies for TNBC. All three treatments inhibited tumor growth but did not induce complete regression (Fig. 1A). We then collected vehicle-treated and PTX-treated tumors and performed high-throughput mRNA quantitation of 770 genes associated with cancer progression using the nCounter analysis system. Among the 236 genes upregulated upon chemotherapy, 57 are mesenchymal-annotated genes (Supplementary Table S1), suggesting the induction of EMT. Since nCounter analysis system cannot quantitatively differentiate between human mRNAs from tumor cells and mouse mRNAs from stromal cells, we used qPCR analysis with human gene-specific primers to confirm that chemotherapies upregulated EMT-inducing transcription factors *SNAI1/2* specifically in residual tumor cells (Fig. 1B).

Among upregulated genes that are related to EMT, we are particularly interested in periostin (POSTN) due to its consistent induction in tumor cells in response to all three types of chemotherapeutic agents using human periostin-specific primers (Fig. 1B). Induction of human periostin was also observed in another human basal-like TNBC model, SUM149, treated with PTX (Supplementary Fig. S1A and B). Furthermore, *POSTN* mRNA expression is significantly correlated with an EMT gene signature in Cancer Cell Line Encyclopedia (CCLE) database (Supplementary Fig. S1C). Among various cancer types, breast cancer cells showed the strongest correlation (Fig. 1C). The known periostin receptor integrin α_V_β_3_ (*ITGAV* and *ITGB3*)^11^ is also correlated with *POSTN* in breast cancer (Fig. 1C). In breast cancer patients, survival analysis showed that high expression of *POSTN* predicted a significantly higher risk of recurrence specifically in basal-like breast cancer (Fig. 1D; Supplementary Fig. S1D), which is largely overlapped with histologically-defined TNBC. Importantly, when we analyzed 26 pairs of chemoresistant (cases with residual disease after neoadjuvant chemotherapy) human TNBC samples before and after chemotherapy, 19 cases showed significant upregulation of periostin at the cancer-stroma interface after chemotherapy (Fig. 1E,F; Supplementary Table S2). Together, these data strongly indicate that induction of periostin is significantly associated with chemoresistance in human TNBC.

**Figure 2:**
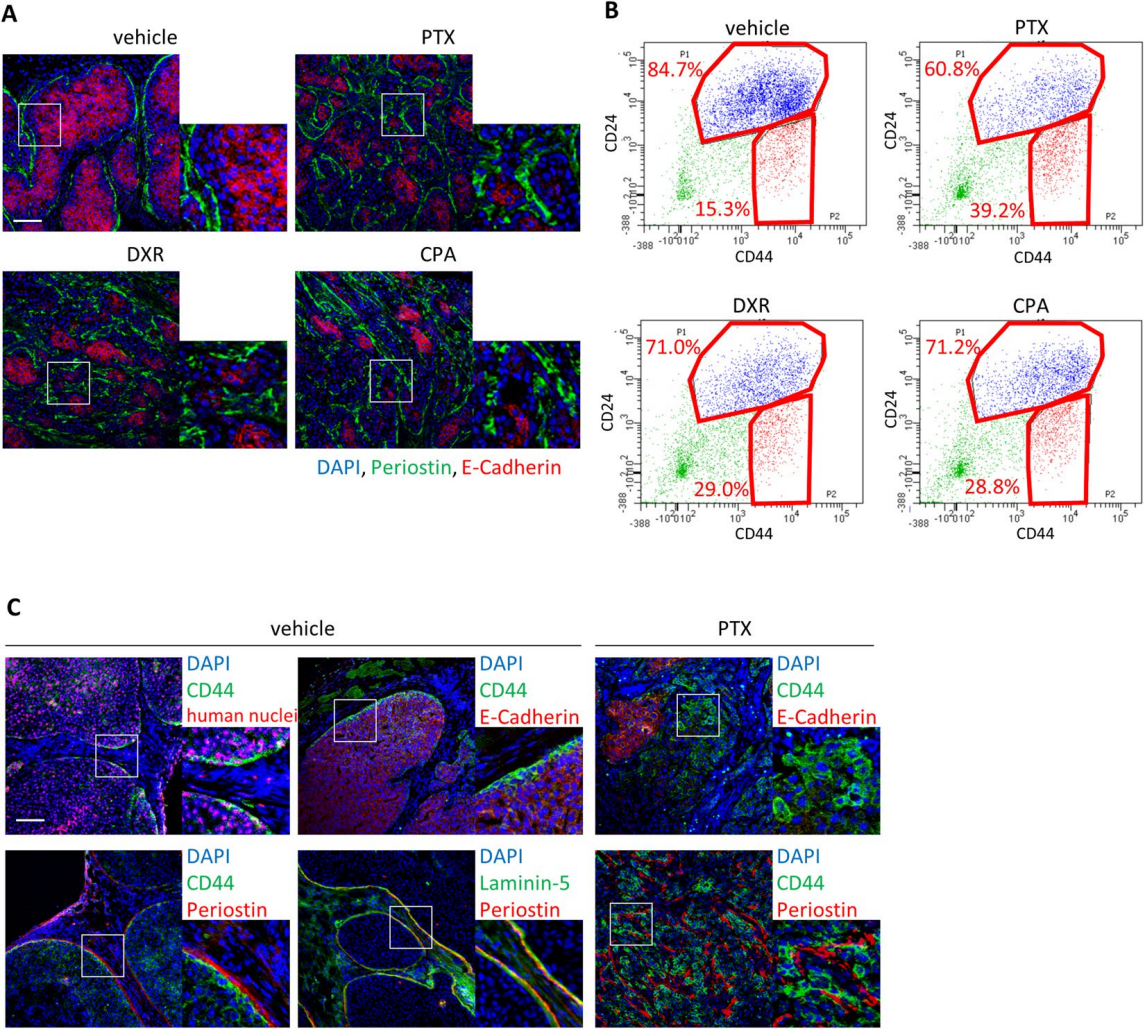
Chemotherapy enriches periostin protein and the mesenchymal cell population in TNBC xenografts. (**A**) Immunofluorescence staining of periostin and E-cadherin in tumors harvested at the end of the experiment. Right lower inserts are enlarged pictures of squared area. Scale bar, 100 µm. (**B**) Flow cytometry analysis of tumors harvested at the end of the experiment using CD44 and CD24 antibodies. Samples were prepared from the mixture of 5 tumors. Ratio of CD44^high^/CD24^low^ to CD24^high^ tumor cells is presented on the plots. (**C**) Immunofluorescence staining of CD44, human nuclei, E-cadherin, periostin, and laminin-5 in tumors harvested at the end of the experiment. Right lower inserts are enlarged pictures of squared area. Scale bar, 100 µm.

We next examined periostin protein and EMT features in residual tumors after chemotherapy in both MCF10DCIS and SUM149 xenografts by immunofluorescence analysis. Before chemotherapy, E-cadherin is present at cell-cell junctions in most tumor cells and periostin, a secreted extracellular protein, is largely localized at the edge of tumor islands; while residual tumors after treatment with paclitaxel (PTX), doxorubicin (DXR) and cyclophosphamide (CPA) showed downregulation of E-cadherin and upregulation of periostin (Fig. 2A; Supplementary Fig. S1E). In breast cancer, CD44^high^/CD24^low^ cell population is known to mark tumor-initiating cells with mesenchymal characteristics^12^. In both MCF10DCIS and SUM149 TNBC tumors, after depleting mouse stromal cells using antibodies against mouse MHC Class I, we observed that the percentage of CD44^high^/CD24^low^ human tumor cell population increased significantly after chemotherapy (Fig. 2B; Supplementary Fig. S1F). In MCF10DCIS tumors, human nuclei^+^/CD44^high^ cancer cells detected by a human-specific CD44 antibody at the tumor-stroma interface were mostly E-cadherin^-^ and associated with periostin at laminin-5^+^ basement membrane, which became prominent after PTX treatment (Fig. 2C). Flow cytometry analysis showed that CD44^high^/CD24^low^ population of MCF10DCIS tumors expressed higher expression levels of EMT genes, human *POSTN*, and integrin α_V_β_3_ (Supplementary Fig. S2A and B). Furthermore, upon PTX treatment, the CD44^high^/CD24^low^ population of MCF10DCIS tumors showed a significant increase of human periostin mRNA expression (Supplementary Fig. S2A), suggesting that human periostin expression is also increased in this population in response to PTX treatment. Furthermore, this population presented several properties of tumor-initiating cells, including higher aldehyde dehydrogenase (ALDH) activity (Supplementary Fig. S2B), higher tumorigenic activity (Supplementary Fig. S2C), and the ability to recapitulate original heterogeneous tumors (Supplementary Fig. S2Ds). Together, these results show that induction of periostin and the EMT program is tightly linked with chemoresistance in human TNBC xenografts and patient samples.

### Chemotherapy triggers mesenchymal cell-specific proliferation and promotes invasive phenotype in residual tumors

**Figure 3:**
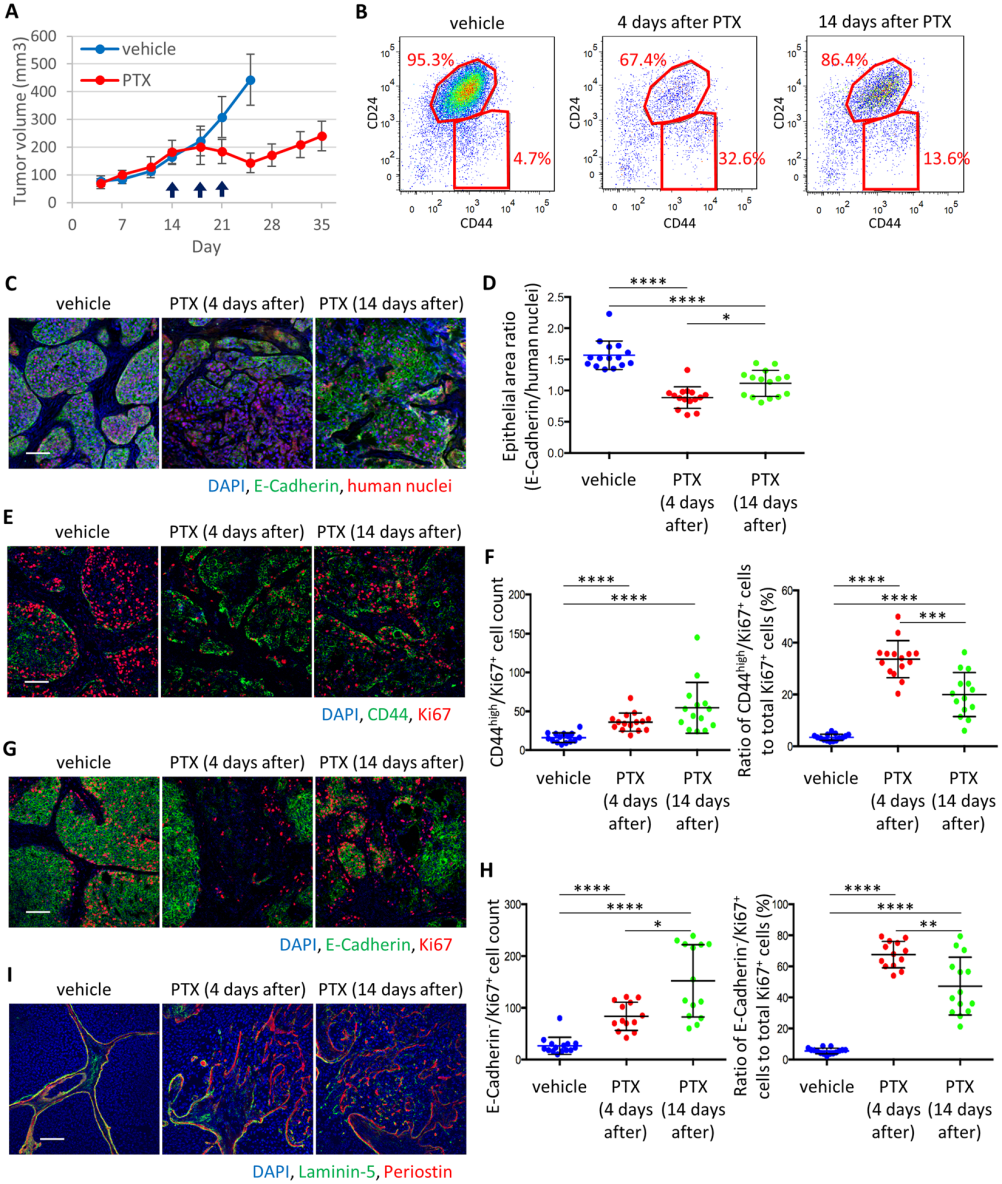
Dynamic regulation of mesenchymal cell expansion and tumor invasion in residual tumors upon chemotherapy. (**A**) Tumor growth curve following relapse from paclitaxel (PTX) treatment in MCF10DCIS xenografts. Arrows indicate times of drug administration. Tumor volume was measured on the indicated days and presented as mean ± standard error (n = 10). (**B**) Flow cytometry analysis of indicated tumors using CD44 and CD24 antibodies. Samples were prepared from the mixture of 10 tumors. Ratio of CD44^high^/CD24^low^ to CD24^high^ tumor cells is presented on the plots. (**C**) Immunofluorescence staining of E-Cadherin and human nuclei in tumor. Scale bar, 100 µm. (**D**) Quantitation of the epithelial area ratio in tumors (n = 15). E-Cadherin positive area was normalized against the total human nuclei positive area. Bars represent mean, and error bars represent standard deviation. *P < 0.05, ****P < 0.0001, Mann-Whitney’s test. (**E**) Immunofluorescence staining of CD44 and Ki67 in tumors. Scale bar, 100 µm. (**F**) The number of CD44^high^/Ki67^+^ cancer cells per field were counted (left panel, n = 15), and the ratio of CD44^high^/Ki67^+^ cancer cells against total Ki67^+^ cells was calculated (right panel, n = 15). Bars represent mean, and error bars represent standard deviation. ***P < 0.001, ****P < 0.0001, Mann-Whitney’s test. (**G**) Immunofluorescence staining of E-Cadherin and Ki67 in tumors. Scale bar, 100 µm. (**H**) The number of E-Cadherin−/Ki67+ cancer cells per field were count (left panel, n = 15), and the ratio of E-Cadherin−/Ki67+ cancer cells against total Ki67+ cells was calculated (right panel, n = 15). Bars represent mean, and error bars represent standard deviation. ***P < 0.001, ****P < 0.0001, Mann-Whitney’s test. (**I)** Immunofluorescence staining of laminin-5 and periostin in tumors. Scale bar, 100 µm.

Next, we examined the dynamics of tumor relapse after PTX treatment using MCF10DCIS xenografts (Fig. 3A). Mouse stromal cells were first depleted using antibodies against mouse MHC Class I during FACS analysis. The CD24^high^ epithelial population first decreased 4 days after chemotherapy and then increased 14 days after chemotherapy by flow cytometry (Fig. 3B). Accordingly, E-cadherin^+^ epithelial area ratio in tumor decreased by PTX treatment and recovered after PTX withdrawal (Fig. 3C,D). Because CD44^high^ cancer cells drastically increased 4 days upon PTX treatment, we checked their proliferation status by Ki67 staining. Although most of CD44^high^ cancer cells were Ki67^−^ in the vehicle-treated tumors, the number and proportion of CD44^high^/Ki67^+^ cancer cells in the tumor significantly increased upon PTX treatment (Fig. 3E,F). We also analyzed E-cadherin^−^/Ki67^+^ cancer cells and found that the number and proportion of CD44^high^/Ki67^+^ cancer cells also increased (Fig. 3G,H). In addition, the epithelial tumors lost its well-encapsulated ductal carcinoma *in situ* (DCIS)-like histology and adopted an invasive phenotype upon PTX treatment. Immunostaining showed disrupted basement membrane labeled with laminin-5 and strong periostin signal in the invasive area (Fig. 3I). Membrane-type metalloproteases (MT-MMPs) are required for the proteolysis of basement membrane *in vivo*^13^. MMP-2 and MMP-9 are known to be proteolytically activated by MT1-MMP and assist cancer cell invasion^14^. Indeed, mRNA expression of human MMP-2, MMP-9 and MMP-14 (MT1-MMP) was upregulated by PTX treatment (Supplementary Fig. S3A) and MT1-MMP protein became abundant in E-cadherin^-^ mesenchymal tumor cells (Supplementary Fig. S3B). Furthermore, gelatin zymography showed that MMP-9 activity was upregulated in PTX-treated tumors (Supplementary Fig. S3C) likely due to the expansion of mesenchymal cancer cell population. Consistently, SUM149 tumors showed similar response to chemotherapy (Supplementary Fig. S3D–J). Together, these data demonstrate that chemotherapy induces upregulation of periostin in tumor cells and expansion of mesenchymal tumor cells with an increased expression and activity of MT-MMPs.

### Periostin knockdown inhibits mesenchymal cell expansion upon chemotherapy and prolonged relapse-free survival

**Figure 4:**
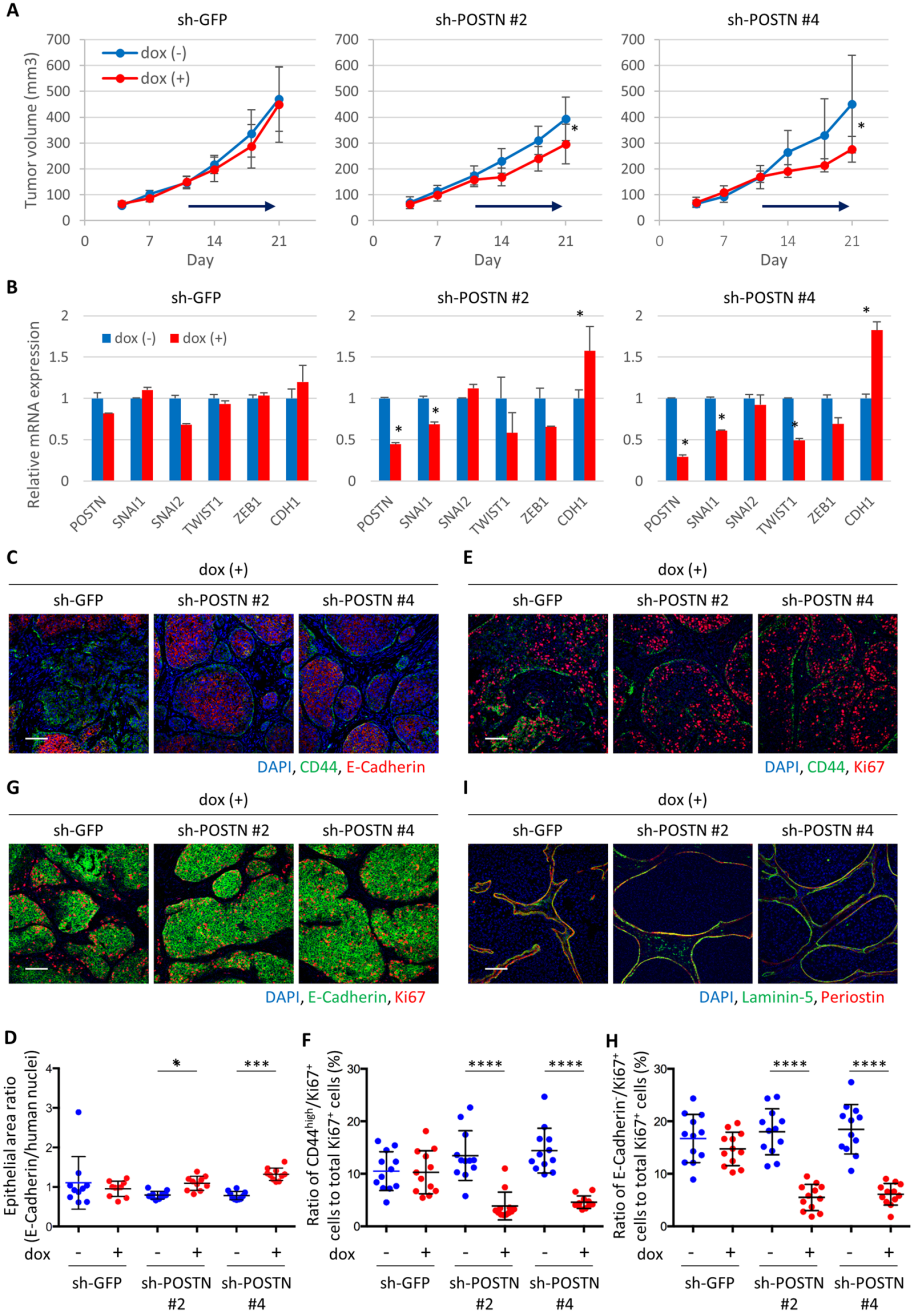
Periostin knockdown inhibited mesenchymal cell proliferation and maintained non-invasive phenotype in TNBC tumors. (**A**) Tumor growth curve in MCF10DCIS tumors upon dox-inducible knockdown of periostin. Arrow indicates administration period of dox. Tumor volume was measured on the indicated days. Data are presented as mean ± standard error (n = 10). *P < 0.05, Student’s *t* test. (**B**) Quantitative PCR analysis of indicated genes in tumors collected at the end of the experiment. Samples were prepared from a mixture of 10 tumors. Relative values are presented as mean ± standard error, *P < 0.02, Student’s *t* test. (**C**) Immunostaining of CD44 and E-cadherin in tumors collected at the end of the experiment. Scale bar, 100 µm. (**D**) Quantitation of the epithelial area ratio in tumors collected at the end of the experiment (n = 10). E-cadherin positive area was normalized against the total human nuclei positive area. Bars represent mean, and error bars represent standard deviation. *P < 0.05, ***P < 0.001, Mann-Whitney’s test. (**E**) Immunostaining of CD44 and Ki67 in tumors collected at the end of the experiment. Scale bar, 100 µm. (**F**) The ratio of CD44^high^/Ki67^+^ cancer cells against total Ki67^+^ cells was calculated (n = 12). Bars represent mean, and error bars represent standard deviation. ****P < 0.0001, Mann-Whitney’s test. (**G)** Immunostaining of E-cadherin and Ki67 in tumors collected at the end of the experiment. Scale bar, 100 µm. (**H**) The ratio of E-cadherin^−^/Ki67^+^ cancer cells against total Ki67^+^ cells was calculated (n = 12). Bars represent mean, and error bars represent standard deviation. ****P < 0.0001, Mann-Whitney’s test. (**I**) Immunostaining of laminin-5 and periostin in tumors collected at the end of the experiment. Scale bar, 100 µm.

To understand the role of periostin *in vivo*, we knocked down human periostin in MCF10DCIS tumor cells using a doxycycline (dox)-inducible shRNA system. Induction of human periostin knockdown delayed tumor growth in mice (Fig. 4A). Consistent with gene expression changes indicating a decrease of the mesenchymal population (Fig. 4B), immunostaining showed that human periostin knockdown reduced CD44^high^/E-cadherin^−^ cell region and increased epithelial cell population (Fig. 4C,D). The ratio of CD44^high^/Ki67^+^ and E-cadherin^−^/Ki67^+^ cells were also reduced by periostin knockdown (Fig. 4E–H). Although currently available periostin antibodies all recognize both human and mouse species of periostin, immunostaining showed significantly reduced periostin signal in the tumors with human periostin knockdown (Fig. 4I). More importantly, laminin-5 staining showed that human periostin knockdown resulted in tumors with a more intact basement membrane and DCIS-like histology (Fig. 4I).

**Figure 5:**
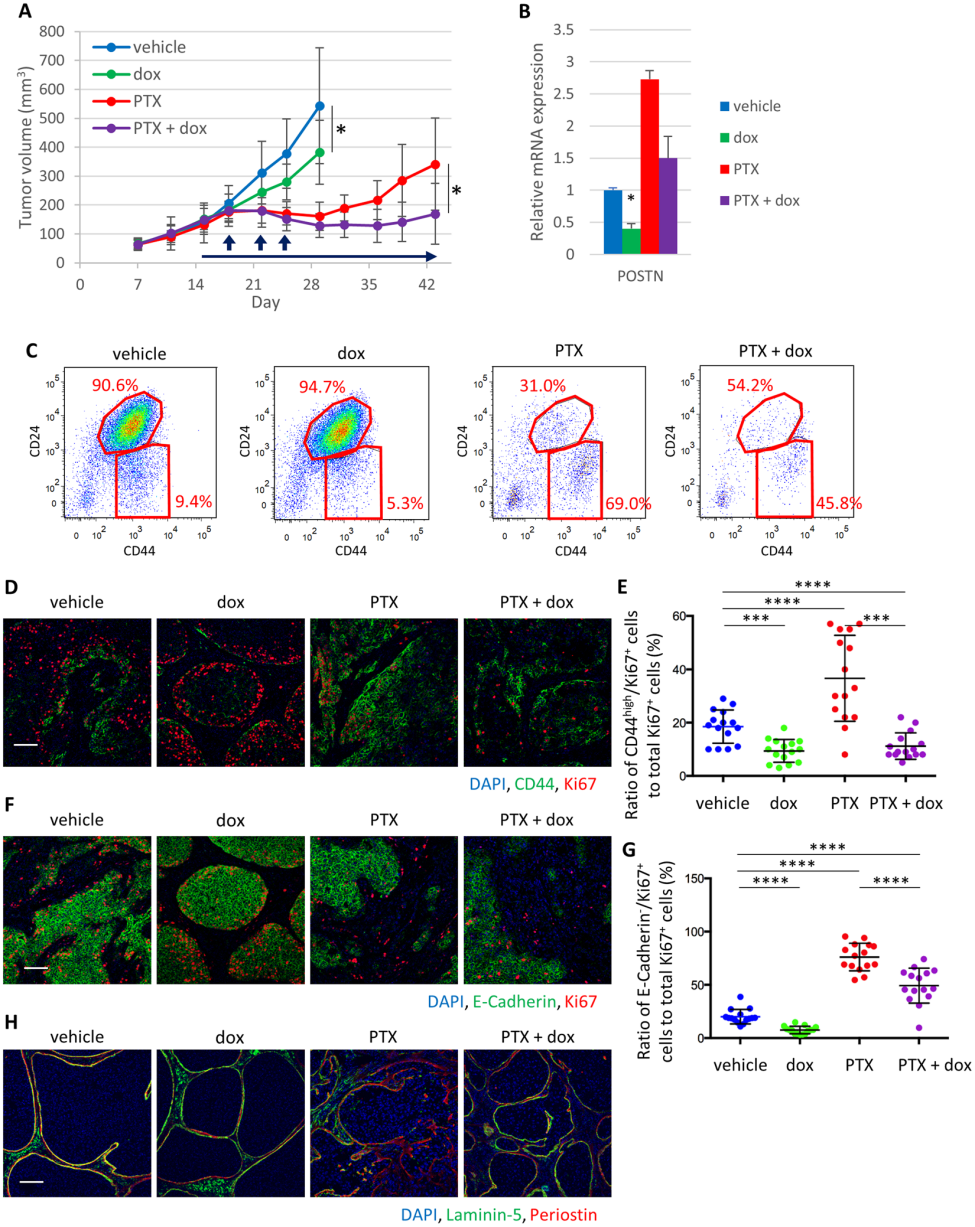
Periostin is required for mesenchymal cell expansion and tumor invasion in residual tumors upon chemotherapy. (**A**) Tumor growth curve in MCF10DCIS xenografts upon PTX treatment and periostin knockdown. Vertical arrows indicate times of PTX administration, and horizontal arrow indicates administration period of doxycycline to knock down periostin. Tumor volume was measured on the indicated days and are presented as mean ± standard error (n = 10). *P < 0.05, Student’s *t* test. (**B**) Quantitative PCR analysis of *Periostin* gene in tumors collected at Day 30. Samples were prepared from a mixture of 10 tumors. Relative values are presented as mean ± standard error, *P < 0.02, Student’s *t* test. (**C**) Flow cytometry analysis of tumors at Day 30 using CD44 and CD24 antibodies. Samples were prepared from the mixture of 10 tumors. Ratio of CD44^high^/CD24^low^ to CD24^high^ tumor cells is presented on the plots. (**D**) Immunofluorescence staining of CD44 and Ki67 in tumors collected at the end of the experiment. Scale bar, 100 µm. (**E**) Ratio of CD44^high^/Ki67^+^ cancer cells against total Ki67^+^ cells was quantified at Day 30 (n = 15). Bars represent mean, and error bars represent standard deviation. ***P < 0.001, ****P < 0.0001, Mann-Whitney’s test. (**F**) Immunofluorescence staining of E-cadherin and Ki67 in tumors collected at the end of the experiment. Scale bar, 100 µm. (**G**) The ratio of E-cadherin−/Ki67+ cancer cells against total Ki67+ cells was calculated (n = 12). Bars represent mean, and error bars represent standard deviation. ****P < 0.0001, Mann-Whitney’s test. (**H**) Immunofluorescence staining of laminin-5 and periostin in tumors collected at the end of the experiment. Scale bar, 100 µm.

We then performed chemotherapy treatment in combination with periostin knockdown. After primary tumors are formed, we started doxycycline treatment to induce the shRNA against human periostin three days prior to chemotherapy. The combination treatment suppressed tumor relapse after PTX treatment (Fig. 5A,B). Importantly, knockdown of periostin reversed many cellular and molecular changes induced by chemotherapy: reduction of the mesenchymal cell population identified as being CD44^high^/CD24^low^, CD44^high^/E-cadherin^−^, or CD44^high^/vimentin^+^ cells (Fig. 5C; Supplementary Fig. S4A), decrease of chemotherapy-induced proliferation of mesenchymal tumor cells (Fig. 5D–G). Immunostaining revealed that human periostin knockdown led to tumors with more intact basement membrane as indicated by laminin-5 staining(Fig. 5H) and reduced expression of MT1-MMP (Supplementary Fig. S4B). MMP-9 and MT1-MMP mRNA expression and MMP-9 activity were also reversed by periostin knockdown (Supplementary Fig. S4C and D). Together, these data indicate that periostin in tumor cells is functionally important for chemotherapy induced mesenchymal tumor cell expansion, which consequently causes disruption of basement membrane and invasion.

### Periostin promotes mesenchymal cell proliferation upon chemotherapy

**Figure 6:**
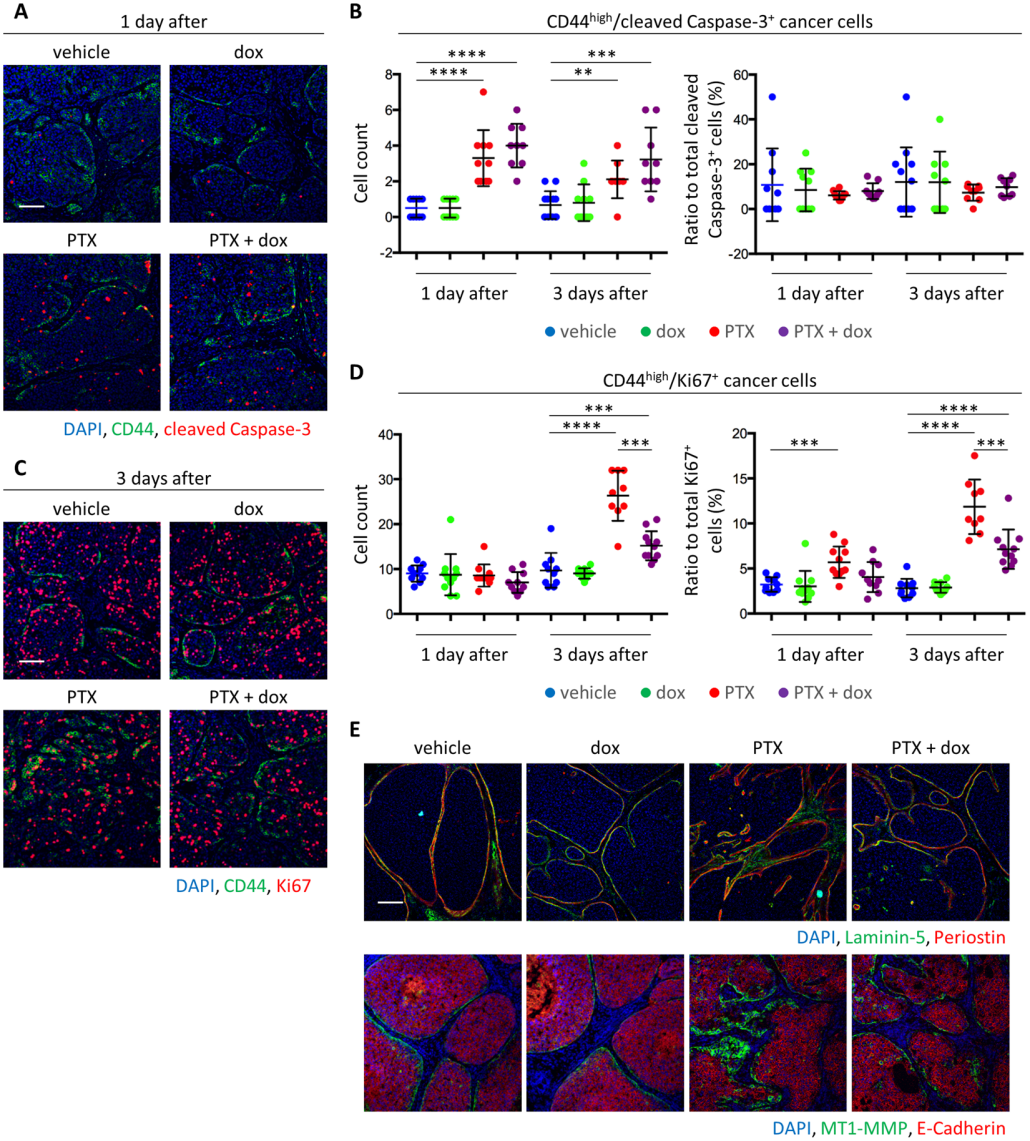
Knockdown of periostin inhibited mesenchymal cell proliferation and invasion, but did not affect apoptosis induction immediately after chemotherapy. (**A**) Immunofluorescence staining of CD44 and cleaved caspase-3 in MC10DCIS tumors. Tumors were sampled 1 or 3 days after one dose of PTX treatment. Scale bar, 100 µm. (**B**) The number of CD44^high^/cleaved Caspase-3^+^ cancer cells per field were counted (left panel, n = 10), and the ratio of CD44^high^/cleaved caspase-3^+^ cancer cells against total cleaved caspase-3^+^ cells was calculated (right panel, n = 10). Bars represent mean, and error bars represent standard deviation. **P < 0.01, ***P < 0.001, ****P < 0.0001, Mann-Whitney’s test. (**C**) Immunofluorescence staining of CD44 and Ki67 in MC10DCIS tumors. Tumors were sampled 1 or 3 days after one dose of PTX treatment. Scale bar, 100 µm. (**D**) The number of CD44^high^/Ki67^+^ cancer cells per field were counted (left panel, n = 10), and the ratio of CD44^high^/Ki67^+^ cancer cells against total Ki67^+^ cells was calculated (right panel, n = 10). Bars represent mean, and error bars represent standard deviation. ***P < 0.001, ****P < 0.0001, Mann-Whitney’s test. (**E**) Immunofluorescence staining of laminin-5, periostin, E-cadherin and MT1-MMP in tumors. Tumors were sampled 3 days after one dose of PTX treatment. Scale bar, 100 µm.

To understand how periostin confers chemoresistance in mesenchymal cancer cells, we analyzed tumors one or three days after one dose of PTX treatment. As expected, the number of apoptotic cancer cells in CD44^high^ population increased 1 day after PTX treatment. Surprisingly, chemotherapy in combination with periostin knockdown did not significantly affect the number of apoptotic cells (Fig. 6A,B). There was also no change in the ratio of CD44^high^/cleaved caspase-3^+^ cancer cells against total cleaved caspase-3^+^ cells (Fig. 6B). In contrast, cell proliferation in the CD44^high^ cancer cells rose significantly 3 days after PTX treatment, and periostin knockdown significantly suppressed this increase (Fig. 6C,D). Similarly, the ratio of E-cadherin^−^/cleaved caspase-3^+^ cancer cells was not affected by the combination, but the ratio of E-cadherin^−^/Ki67^+^ cancer cells was reduced by the combination (Supplementary Fig. S4E and F). Destabilization of basement membrane and MT1-MMP upregulation was also observed 3 days after chemotherapy and periostin knockdown reversed the invasive phenotype (Fig. 6E).

Consistent with the *in vivo* data, we analyzed *POSTN* expression in immortalized human mammary epithelial HMLE cells that have undergone EMT in response to expression of SNAIL1 or TWIST1. Both HMLE/SNAIL1 and HMLE/TWIST1 cells showed upregulation of periostin mRNA and protein (Supplementary Fig. S5A and B). Periostin knockdown did not affect E-cadherin and vimentin expression in HMLE/TWIST1 cells (Supplementary Fig. S5C), while it specifically inhibited cell proliferation of HMLE-SNAIL1 and HMLE-TWIST1 cells, but not control cells in 2D culture (Supplementary Fig. S5D). Moreover, periostin knockdown also suppressed cell invasion in transwell assays and 3D organoid culture only in HMLE-SNAIL1 and HMLE-TWIST1 cells (Supplementary Fig. S5E–H). Together, these data show that periostin is specifically required for proliferation and invasion, but not for anti-apoptosis function of mesenchymal tumor cells in response to chemotherapy.

### A functional blocking antibody targeting cancer-specific periostin variants inhibits invasive relapse after chemotherapy

**Figure 7:**
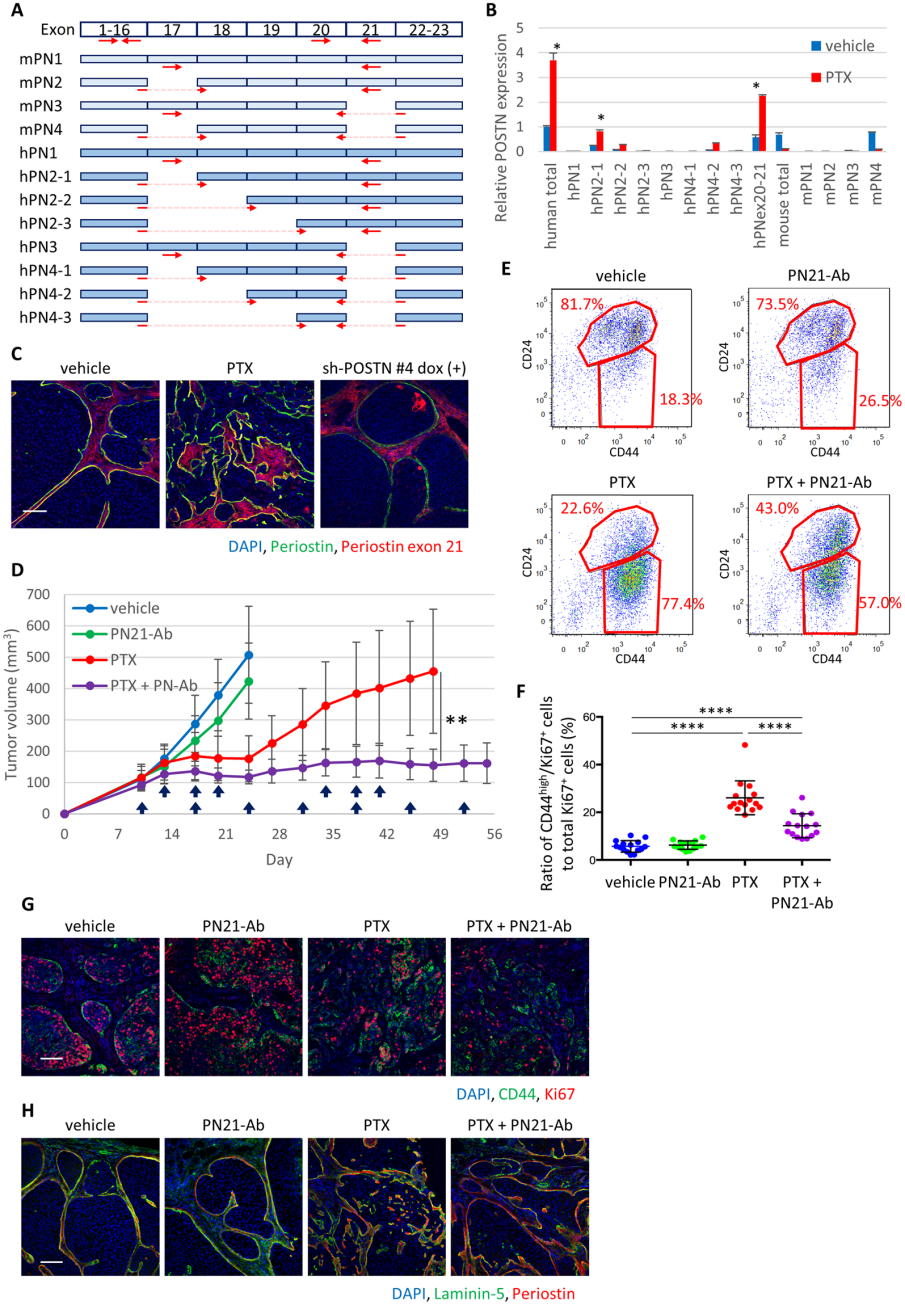
An antibody targeting cancer-specific periostin variants inhibited mesenchymal cell expansion and tumor invasion after chemotherapy and halted tumor progression. (**A**) Diagram of splicing variants of human and mouse *POSTN*. Arrows indicate the location of designed primers. (**B**) Quantitative PCR analysis of *POSTN* splicing variants in tumor with or without PTX treatment. The region over exon 20 and 21 (hPNex20-21) was also amplified. Relative values against human total *POSTN* expression in vehicle-treated tumor are presented. Relative values are presented as mean ± standard error, *P < 0.02, Student’s *t* test. (**C**) Immunofluorescence staining of periostin variants containing exon 21 in tumor upon PTX treatment and periostin knockdown. Scale bar, 100 µm. (**D**) Tumor growth curve in MCF10DCIS xenografts treated with PTX and/or PN21-Ab. Upper arrows indicate times of PTX administration, and lower arrows indicate times of PN21-Ab administration. Tumor volumes were measured on the indicated days and are presented as mean ± standard error (n = 10). **P < 0.01, Student’s *t* test. (**E**) Flow cytometry analysis of tumors at Day 24 using CD44 and CD24 antibodies. Samples were prepared from a mixture of 10 tumors. Ratio of CD44^high^/CD24^low^ to CD24^high^ tumor cells is presented on the plots. (**F**) The ratio of CD44^high^/Ki67^+^ cancer cells against total Ki67^+^ cells was quantified in tumors collected at Day 24 (n = 15). Bars represent mean, and error bars represent standard deviation. ****P < 0.0001, Mann-Whitney’s test. (**G**) Immunofluorescence staining of CD44 and Ki67 in tumors collected at Day 24. Scale bar, 100 µm. (**H**) Immunofluorescence staining of laminin-5 and periostin in tumors collected at Day 24. Scale bar, 100 µm.

Four splicing variants of mouse periostin and eight splicing variants of human periostin have been reported and characterized^15–17^. PN4 is the most abundant variant in normal tissue, and other variants are known to be induced in various inflammatory diseases including cancer. Periostin knockout mice showed abnormal bone development with severe somatic growth retardation^18^. Considering these observations, we first examined the expression of mouse and human periostin variants in MCF10DCIS tumor with or without chemotherapy using variant-specific primer sets (Fig. 7A) and found that hPN2-1 and hPN2-2 were the most dominant variants after PTX-treatment (Fig. 7B). The region over exon 20 and 21 (hPNex20-21), which is characteristic of PN1 and PN2 variants, also showed increased expression. It is also important to note that there is no significant increase of any mouse periostin variants that are expressed in cancer-associated mouse stroma cells, further supporting the notion that periostin is specifically induced in human tumor cells (Fig. 7B). Using a periostin monoclonal antibody specifically against exon 21 region (PN21-Ab), which does not recognize PN4, we found that PTX treatment significantly increased the expression of periostin variants containing exon 21, which can be suppressed by periostin knockdown (Fig. 7C).

PN21-Ab has been characterized as a functional blocking antibody in mice^19^, therefore we combined PN21-Ab treatment with chemotherapy in TNBC xenografts. Importantly, PN21-Ab treatment significantly suppressed tumor relapse after PTX treatment (Fig. 7D). PN21-Ab treatment did not enhance the toxicity of PTX in body weight (Supplementary Fig. S6A) or somatic growth as indicated by tail length measurement (Supplementary Fig. S6B). The combination treatment also partially reversed the increase of CD44^high^/CD24^low^ population upon PTX treatment (Fig. 7E), and decreased mesenchymal tumor cell proliferation (Fig. 7F,G; Supplementary Fig. S6D–E). Immunofluorescence analysis revealed that periostin knockdown partially reversed PTX-induced invasive phenotype (Fig. 7H) and MT1-MMP upregulation in E-cadherin^-^ cancer cells (Supplementary Fig. S6F). The results strongly suggest that pharmacological inhibition of cancer-specific variants of periostin can effectively suppress mesenchymal tumor cell expansion and overcome chemoresistance.

## Discussion

Early relapse after chemotherapy poses a significant challenge in the treatment of patients with TNBC. The ability of tumor cells to undergo alternative cell fates, such as cellular senescence, epithelial-mesenchymal plasticity, and stem-cell reprograming, is the main driver of acquired resistance against therapies that target only a single cellular phenotype^20^. Therefore, regulating one of these processes, in addition to chemotherapy, may tip the balance to favor a durable treatment response and improved long-term survival in patients with TNBC.

Here, we show that periostin promotes proliferation and expansion of a resilient subpopulation of mesenchymal tumor cell population that survived chemotherapy treatment, thus suggesting a critical role of periostin in expanding the mesenchymal cell population and leading to chemoresistance and tumor relapse. Using *in vivo* xenograft model of TNBC, we clearly show that inhibition of periostin, in combination with chemotherapy, led to a more durable chemotherapy response by restricting the expansion of the residual mesenchymal tumor cell population. Antibody targeting cancer-specific variants of periostin also halted disease progression after chemotherapy without toxicity in mice, suggesting its potential as a therapeutic agent to combat chemoresistance.

Our observation that proliferation of mesenchymal population contributes to chemoresistance of TNBC is in agreement with a recent report where TNBC PDX tumors were found to develop resistance upon continuous exposure to taxanes due to the expansion of a pre-existing CD49f+ chemoresistant population with tumor initiating capability but without stem-cell phenotype^21^. Additional clinical studies also showed that residual breast cancers after chemotherapy displayed mesenchymal and tumor-initiating features^12,22,23^. The exact mechanism of how periostin leads to mesenchymal tumor cell expansion is unknown. Periostin can bind to and activate Integrin α_V_β_3_ to activate FAK and AKT signaling to enhance proliferation^11^. Periostin is also known to modify the ECM and could then increase proliferation via modifying other ECM-mediated signaling from the tumor microenvironment^24,25^. Further understanding of the key downstream targets of periostin in mesenchymal cell proliferation may identify new targets against chemoresistance.

Together, our findings suggest that periostin blockade in combination with chemotherapy can be an effective therapeutic approach to improve survival in patients with TNBC. Given that the involvement of periostin in cancer stem cell functions, metastasis and chemoresistance is observed in other human cancers^26–29^, this strategy could prove to be effective in overcoming chemoresistance beyond triple-negative breast cancer.

## Methods

### Human cancer database analysis

The dataset of mRNA expression in cancer cells was downloaded from the Cancer Cell Line Encyclopedia (CCLE) website (http://www.broadinstitute.org/ccle). Heatmap was generated in Excel (Microsoft), and Pearson product-moment correlation coefficient was calculated. Breast cancer patient survival was examined using online meta-analysis tool (http://kmplot.com/analysis/). Patients were stratified by *POSTN* (Affy ID: 210809_s_at) expression, and recurrence-free survival in each group was analyzed.

### Cell culture

MCF10DCIS cells were cultured in DMEM/F12 media with 5% horse serum, 20 ng/ml EGF, 10 μg/ml insulin, 0.5 μg/ml hydrocortisone, and 100 ng/ml cholera toxin. SUM149 cells were cultured in F12 media with 5% fetal bovine serum, 5 μg/ml insulin, 1 μg/ml hydrocortisone, and 10 mM HEPES. HMLE cells were cultured in MEGM (Lonza) mixed 1:1 with DMEM/F12 media with 10 ng/ml EGF, 10 μg/ml insulin, 0.5 μg/ml hydrocortisone. All cell lines were routinely tested to be free from mycoplasma and authenticated by short tandem repeat profiling.

### 3D organoid culture

Single suspended cells were seeded on growth factor-reduced Matrigel (GFR-Matigel, BD Biosciences) in 2% GFR-Matrigel MCF10DCIS media. For the evaluation of cancer invasion, non-invasive and invasive acini were count on at least 10 bright-field images. Invasive acini were scored as acini that adopted a spread and invasive phenotype.

### Generation of stable cell lines

Stable cell lines were generated using retroviral or lentiviral plasmid vectors. Briefly, concentrated viral supernatants were applied to target cells with 6 μg/ml protamine sulphate. Infected cells were then selected for with puromycin or blasticidin. TWIST1 and SNAIL1 cDNAs were cloned into the pWZL-Blast vector. The shRNAs targeting *GFP* (5′-CGTGATCTTCACCGACAAGAT-3′) and *POSTN* (#2: 5′-CGGTGACAGTATAACAGTAAA-3′ (exon 8), #4: 5′-GCAGAGAAATCCCTCCATGAA-3′ (exon 11)) were cloned into the pLKO-Tet-On vector (Addgene).

### Transwell invasion assay

*In vitro* invasion were evaluated with 24-well Transwell Permeable Supports (8 μm pore size, Coster). Briefly, a total of 40,000 cells were cultured on GFR-Matrigel-coated Transwell inserts for 48 hours. The top membrane was cleaned and washed and the inserts were fixed with 4% paraformaldehyde (PFA) and stained with 0.1% crystal violet. Crystal violet was released with 10% acetic acid, and the absorbance was measured at 540 nm.

### *In vivo* mouse experiment

All animal care and studies were performed in accordance of protocols approved by the Institutional Animal Care and Use Committee of the University of California, San Diego. MCF10DCIS and SUM149 cells (5 × 10^6^) suspended in 50% Matrigel were injected bilaterally into the franks of 7-week-old female BALB/c nude mice. When the tumor volume reached 100 mm^3^, paclitaxel (Sagent Pharmaceuticals, 40 mg/kg), doxorubicin (Sagent Pharmaceuticals, 10 mg/kg) or cyclophosphamide (Sandoz Inc., 300 mg/kg) were injected intravenously three times in 8 days. In some experiments, periostin antibody (PN21-Ab, 10 mg/kg) was injected intravenously once a week in combination with PTX. The tumor volume was calculated by using the following formula: tumor volume (mm^3^) = 1/2 × length (mm) × width (mm)^2^. For *in vivo* knock-down experiment, mice were administered 2 mg/ml doxycycline (Sigma) in drinking water. Toxicity of each treatment was evaluated by body weight, tail length and gross appearance of body.

### Immunostaining analysis

For histological analysis, tumors were embedded in OCT compound (Sakura), and cryosections were prepared. Sections were fixed with methanol and stained with antibodies against periostin (GeneTex, GTX100602), periostin exon 21 (PN21-Ab), E-cadherin (BD Biosicences, 610181 and Cell Signaling Technologies, 9835), human nuclei (Millipore, MAB1281C3), CD44 (Cell Signaling Technologies, 4041), vimentin (GeneTex, GTX100619), laminin-5 (Millipore, MAB19562), Ki67 (Pierce, PA5-19462), cleaved caspase-3 (Cell Signaling Technologies, 9661) and MT1-MMP (GeneTex, GTX61603) and Alexa Fluor-conjugated secondary antibodies and streptavidin (ThermoFisher). The staining was performed using M.O.M. Detection kit (Vector Laboratories). To calculate of epithelial area ratio in tumor, E-cadherin or human nuclei fluorescence area was quantitated in at least 3 view fields per tumor by using ImageJ software. For the analysis of cell proliferation or apoptosis in tumor, CD44^high^, Ki67^+^, E-cadherin^+^ or cleaved caspase-3^+^ cells were counted manually in at least 3 view fields per tumor.

### Flow cytometry analysis

Tumors were enzymatically digested with Accumax (Stemcell Technologies). Single cell suspension was stained with antibodies against mouse MHC Class I (eBioscience, 11-5998), human-specific CD44 (BD Bioscience, 555479), CD24 (BioLegend, 311105) and Integrin α_V_β_3_ (Millipore, MAB1976X) and Aldefluor kit (Stemcell Technologies) and analyzed by using a FACS Aria system (BD Bioscience). Cells that are positive for propidium iodide (Sigma-Aldrich) and negative for mouse MHC staining were selected as live human cancer cells. Cancer cells were sorted from the mixture of all the tumors in each treatment group and applied to tumorigenicity assay and quantitative PCR analysis.

### Gene expression analysis

RNA was extracted from cells or tumors using the RNeasy Mini and Micro Kit (Qiagen), and cDNA was generated with a cDNA Reverse Transcription Kit (Applied Biosystems). Real-time quantitative PCR was carried out with a CFX Connect system (Bio-Rad). Expression values were generated using ddCt method normalized by human *HPRT1* expression. The following human primer sets were used: *HPRT1* (5′-CTTGATGATCTCGCGGAATA-3′, 5′-GCATTGTTTTGCCAGTGTCAA-3′), *POSTN* (5′-TACAACGGGCAAATACTGGA-3′, 5′-CTTGATGATCTCGCGGAATA-3′), *POU5F1* (5′-GTGTTCAGCCAAAAGACCATCT-3′, 5′-GGCCTGCATGAGGGTTTCT-3′), *NANOG* (5′-TTTGTGGGCCTGAAGAAAACT-3′, 5′-AGGGCTGTCCTGAATAAGCAG-3′), *SOX2* (5′-GCCGAGTGGAAACTTTTGTCG-3′, 5′-GGCAGCGTGTACTTATCCTTCT-3′), *SNAI1* (5′-AAGATGCACATCCGAAGCC-3′, 5′-CGCAGGTTGGAGCGGTCAGC-3′), *SNAI2* (5′-AAGCATTTCAACGCCTCCAAA-3′, 5′-GGATCTCTGGTTGTGGTATGACA-3′), *TWIST1* (5′-AAGAGGTCGTGCCAATCAG-3′, 5′-GGCCAGTTTGATCCCAGTAT-3′), *ZEB1* (5′-GATGATGAATGCGAGTCAGATGC-3′, 5′-ACAGCAGTGTCTTGTTGTTGTAG-3′), *CDH1* (5′-TGCCCAGAAAATGAAAAAGG-3′, 5′-GTGTATGTGGCAATGCGTTC-3′), *CDH2* (5′-ACAGTGGCCACCTACAAAGG-3′, 5′-CCGAGATGGGGTTGATAATG-3′), *FN1* (5′-TCCCTCGGAACATCAGAAAC-3′, 5′-CAGTGGGAGACCTCGAGAAG-3′), *VIM* (5′-GAGAACTTTGCCGTTGAAGC-3′, 5′-GCTTCCTGTAGGTGGCAATC-3′), *MMP2* (5′-CTTCCAAGTCTGGAGCGATGT-3′, 5′-TACCGTCAAAGGGGTATCCAT-3′), *MMP9* (5′-TGTACCGCTATGGTTACACTCG-3′, 5′-GGCAGGGACAGTTGCTTCT-3′) and *MMP14* (5′-GAAGCCTGGCTACAGCAATATG-3′, 5′-TGCAAGCCGTAAAACTTCTGC-3′). The primer sets for periostin variant analysis were as follows: human total *POSTN* (5′-TACAACGGGCAAATACTGGA-3′, 5′-CTTGATGATCTCGCGGAATA-3′), hPN1 (5′-GTGATTGAAGGCAGTCTTCAGCC-3′, 5′-CTCCCTGAAGCAGTCTTTTA-3′), hPN2–1 (5′-AATCCCCGTGACTGTCTATAGACC-3′, 5′-CTCCCTGAAGCAGTCTTTTA-3′), hPN2–2 (5′-AATCCCCGTGACTGTCTATAAGCCA-3′, 5′-CTCCCTGAAGCAGTCTTTTA-3′), hPN2-3 (5′-AATCCCCGTGACTGTCTATAGTCCT-3′, 5′-CTCCCTGAAGCAGTCTTTTA-3′), hPN3 (5′-GTGATTGAAGGCAGTCTTCAGCC-3′, 5′-TCCTCACGGGTGTGTCTTCT-3′), hPN4-1 (5′-AATCCCCGTGACTGTCTATAGACC-3′, 5′-TCCTCACGGGTGTGTCTTCT-3′), hPN4-2 (5′-AATCCCCGTGACTGTCTATAAGCCA-3′, 5′-TCCTCACGGGTGTGTCTTCT-3′), hPN4-3 (5′-AATCCCCGTGACTGTCTATAGTCCT-3′, 5′-TCCTCACGGGTGTGTCTTCT-3′), hPNex20-21 (5′-CACTAGGATTTCTACTGGAGG-3′, 5′- CTCCCTGAAGCAGTCTTTTA-3′), mouse total *POSTN* (5′-TGGTATCAAGGTGCTATCTGCG-3′, 5′-AATGCCCAGCGTGCCATAA-3′), mPN1 (5′-ATAACCAAAGTCGTGGAACC-3′, 5′-TGTCTCCCTGAAGCAGTCTT-3′), mPN2 (5′-CCATGACTGTCTATAGACCTG-3′, 5′-TGTCTCCCTGAAGCAGTCTT-3′), mPN3 (5′-ATAACCAAAGTCGTGGAACC-3′, 5′-TTTGCAGGTGTGTCTTTTTG-3′) and mPN4 (5′-CCCCATGACTGTCTATAGACC-3′, 5′-TTCTTTGCAGGTGTGTCTTTT-3′). For high-throughput direct mRNA counting, the nCounter PanCancer Progression Panel (NanoString) was utilized according to manufacturer’s instructions.

### Western blot analysis

Cell lysates were prepared with RIPA buffer, electrophoresed and blotted onto PVDF membranes. The membranes were incubated with primary antibodies against GAPDH (GeneTex, GTX100118), periostin (GeneTex, GTX100602), E-cadherin (BD Bioscience, 610181) and vimentin (GeneTex, GTX100619) and secondary antibodies for ECL analysis.

### Cell growth inhibition assay

Cells (10^3^ cells/well) were seeded on 96-well culture plates. After several days culture, TetraZ Cell Proliferation Kit (Biolegend) was used to measure the live cell number.

### Gelatin zymography assay

Tumor lysates were prepared from the mixture of all the tumors in each treatment group with 1% Nonidet P-40 and mixed with SDS loading buffer. Samples were analyzed using Novex 10% gelatin Zymogram gels (ThermoFisher). Gels were first incubated in Zymogram Renaturing Buffer (ThermoFisher) and then incubated in Zymogram Developing Buffer (ThermoFisher).

### Breast cancer tissues

The tissue samples were collected from primary triple-negative breast cancer patients at Osaka National Hospital. 33 patients were treated with neoadjuvant chemotherapy. Tumor specimens were obtained before chemotherapy with vacuum-assisted core needle biopsy (Mammotome). Breast conserving surgery or mastectomy was conducted, and surgical specimens were obtained. Therapeutic effect of chemotherapy was assessed histologically. All patients provided written informed consent. This study was approved by the ethical committee of Osaka National Hospital and conducted in accordance with the Ethical Guideline for Epidemiological Research in Japan.

### Immunohistochemistry analysis

From formalin-fixed, paraffin-embedded tissue samples, 3 μm-thick sections were serially cut and mounted on precoated slides. All procedures were performed automatically in the BenchMark (Ventana Medical Systems). In brief, antigen retrieval was carried out by heating the samples in a CC2 solution at 100 °C for 8 min. Endogenous peroxidase activity was quenched by immersion in 3% hydrogen peroxide for 4 minutes. The tissue sections were incubated with the periostin antibody (Adipogen) for 32 minutes at room temperature. Detection was performed using the LSAB Ventana Iview DAB detection system, according to the manufacturer’s instructions, and sections were counterstained with Hematoxylin. In each sample, we selected 3 representative spots, where tumor and stroma equally existed. We quantitated the area of DAB by ImageJ software and calculated the stain score by averaging them.

### Statistics

Mann-Whitney’s test was performed for comparing multiple treatment groups. For statistical analysis of survival curves of breast cancer patients, log-rank test was performed. For statistical analysis of expression change of periostin after chemotherapy, Wilcoxon signed-rank test was performed. For statistical analysis of qPCR results, two-tailed Student’s *t* test was performed.

### Data availability

All data generated or analysed during this study are included in this published article and its Supplementary Information files.

## Electronic supplementary material

Supplementary Information
