# Supplementary material for: Periostin blockade overcomes chemoresistance via restricting the expansion of mesenchymal tumor subpopulations in breast cancer
Source: Sci Rep. 2018 Mar 5;8:4013. doi: 10.1038/s41598-018-22340-7 (PMC5838092; doi:10.1038/s41598-018-22340-7)
Supplement: Supplementary file 1 — Supplementary Information [file 41598_2018_22340_MOESM1_ESM.pdf]

Periostin blockade overcomes chemoresistance via restricting the expansion of mesenchymal tumor subpopulations in breast cancer

Youya Nakazawa, Yoshiaki Taniyama, Fumihiro Sanada, Ryuichi Morishita, Shoji Nakamori, Koji Morimoto, Kay T. Yeung, and Jing Yang

Supplementary Table 1

Gene	EMT spectrum	PTX/vehicle 1st	PTX/vehicle 2nd
TIE1		10.01	0.78
COL4A6		6.53	2.72
SULF1		6.34	2.62
FIGF		5.56	2.35
BMPR1B		5.00	2.47
PRF1		5.00	0.63
COL7A1		4.95	2.05
EPAS1		4.60	2.30
NRP2		4.50	2.74
ITM2A	M	4.45	4.71
PTGIS	M	4.45	1.05
ROBO4		4.45	2.59
FLT1		4.45	0.39
EGFL7		4.17	1.08
FN1	M	3.78	3.67
GDF15	E	3.77	4.79
TBX1		3.71	1.34
**POSTN**		3.48	2.52
LAMA1		3.46	1.04
KRT1		3.34	2.75
KISS1		3.34	1.88
FBP1		3.34	1.76
NDNF		3.34	1.41
DCC		3.34	1.32
CNN1		3.34	1.18
SFRP2		3.34	0.84
PPP1R16B		3.34	0.78
CDKN1A		3.24	4.04
SMOC1		3.17	2.58
TNC	M	3.15	2.24
CDH13		3.13	1.86
ANGPT1		3.06	0.86
CXCL11		2.97	3.53
TGFBI		2.95	0.94
MCAM		2.80	2.10
PLAU		2.78	1.28
IBSP		2.78	0.67
MYLK	M	2.70	2.05
LAMA3		2.67	1.74
ITGA3		2.62	1.91
NOTCH1		2.61	2.82
EVI2A	M	2.50	0.82
ANGPTL2	M	2.45	1.32
SERPINE1		2.36	1.13
ID1		2.33	2.12
SERPINF1	M	2.33	1.51
PTK2B		2.32	1.19
C3		2.29	1.00
TLR4		2.29	1.53
COL6A1	M	2.26	1.54
COL18A1		2.25	2.33
NAP1L3	M	2.22	3.53
RUNX1T1	M	2.22	3.53
ZFPM2	M	2.22	1.47
CAMK2A		2.22	4.71
IFNG		2.22	4.71
EMILIN3		2.22	3.53
CADM1		2.22	2.55
PLA2G2A		2.22	2.16
SSTR2		2.22	2.12
ANGPT2		2.22	1.96
BMPER		2.22	1.57
PDPN		2.22	1.18
NID2		2.22	0.59
GDF6		2.22	0.24
MYH11		2.22	0.20
IL18		2.22	1.41
NRP1		2.21	1.54
LAMB3		2.17	1.58
AREG		2.16	1.31
CHI3L1		2.16	1.83
APOD		2.10	2.66
HEG1	M	2.09	1.71
CDH11	M	2.07	1.87
FST		2.05	1.63
FLI1	M	2.04	1.06
ABI3BP		2.04	2.35
COL1A1		2.04	1.85
CXCL10		2.02	4.71

Gene	EMT spectrum	PTX/vehicle 1st	PTX/vehicle 2nd
VCAM1	M	2.00	0.78
IL11		2.00	1.50
PIK3CG		2.00	1.18
CCL5		1.98	0.66
CD36		1.97	0.82
MFAP4	M	1.95	0.88
CEACAM6	E	1.95	2.61
TFPI2		1.95	1.29
KRT14		1.94	2.19
SRPX2		1.93	1.81
LAMC2		1.92	0.84
CLDN1		1.92	1.06
TNFSF10		1.91	2.04
VASH1		1.91	1.50
ITGA11		1.90	1.16
MISP		1.89	1.51
ID4		1.89	2.13
COMP		1.88	0.81
HSPB1		1.87	1.66
VAV2		1.87	1.12
COL4A1		1.86	1.11
COL1A2		1.85	0.46
ZEB2	M	1.85	1.37
IL10RA	M	1.85	1.18
DDR2	M	1.85	1.12
NPR1		1.85	1.31
TPSD1		1.85	1.18
PTPRB		1.85	1.07
THBS2		1.82	1.83
FREM2		1.81	1.65
SELE		1.81	1.96
CSPG4		1.80	1.44
SPOCK3		1.78	3.14
SNAI3		1.78	1.06
P3H2		1.77	1.18
MMRN2		1.76	2.92
GIMAP6	M	1.75	1.26
LHFP	M	1.75	0.92
FGF18		1.75	1.73
RORB		1.75	1.68
EMILIN1		1.75	1.03
JAG1		1.74	1.93
APOE		1.73	1.35
PDGFA		1.71	1.69
FSTL1	M	1.70	1.72
CASP8		1.70	1.35
HSPG2		1.70	1.28
MMP14		1.69	1.48
FBLN1	M	1.67	2.36
WIPF1	M	1.67	1.82
HAPLN1		1.67	1.76
MMP24		1.67	1.01
PIK3R5		1.67	0.67
TYMP		1.66	2.15
COL4A2		1.65	1.10
TIMP1		1.65	1.68
FAP	M	1.64	1.38
ACTG2		1.64	0.81
THBS1		1.63	1.39
COL6A2	M	1.63	1.24
PLXDC1		1.62	1.10
SH2B3		1.61	1.18
CAMK2B		1.61	1.09
PTPRM		1.60	1.64
ZEB1	M	1.59	1.00
ITGA1		1.59	1.47
NRCAM		1.59	1.09
LTBP4		1.58	1.18
SNAI2	M	1.58	2.12
EREG		1.58	1.18
SMAD5		1.58	1.12
MAF	M	1.57	1.25
CD163	M	1.56	1.85
BCAS1	E	1.56	1.04
OLFML2B	M	1.55	1.14
IGFBP4		1.55	1.57
COL6A3		1.53	1.60
ACVR1C		1.53	0.83
CXCL13	M	1.52	1.53

Gene	EMT spectrum	PTX/vehicle 1st	PTX/vehicle 2nd
ICAM1		1.50	1.07
ISL1		1.50	1.60
CX3CL1		1.49	1.05
STAT1		1.48	2.87
RAMP1		1.48	2.65
CCR3		1.48	1.57
ALOX5		1.48	1.05
HUNK		1.48	0.84
CTSK	M	1.48	0.97
DST		1.48	1.08
CEP170	M	1.46	1.06
MS4A6A	M	1.45	1.60
TNS1	M	1.45	1.02
AKAP2	M	1.44	1.28
FBN2		1.44	2.07
SACS	M	1.43	1.19
PMP22	M	1.43	1.54
TNFRSF1A		1.43	1.45
TMPRSS4	E	1.42	1.06
POPDC3		1.42	1.05
NR4A3		1.42	0.78
MET		1.40	0.77
CCL8	M	1.39	1.96
FGFR3	E	1.39	1.85
ITGAM		1.39	2.75
GDF5		1.39	1.57
CCL11		1.39	1.37
SOX2		1.39	0.84
HOXB13		1.39	0.71
PLXND1		1.38	0.87
COL5A2	M	1.38	1.27
HIF1A		1.38	1.36
FXYD6	M	1.38	1.25
WWTR1	M	1.37	1.34
FERMT2	M	1.36	1.03
TCF4	M	1.35	0.92
LAMC1		1.35	1.26
OAS1		1.35	2.49
TWIST1	M	1.35	0.92
S1PR1		1.35	0.61
PPFIBP2		1.35	1.15
PTK6	E	1.35	1.06
SRC		1.34	1.14
JUN		1.34	1.20
SPARC	M	1.34	1.93
LAMA5		1.34	1.25
C1S	M	1.33	1.50
HLA-DPB1		1.33	3.19
IL6		1.33	1.88
BMP7		1.33	1.59
TAL1		1.33	1.03
ITGA8		1.33	0.71
MMP17		1.33	0.59
S100A7		1.33	0.97
NR3C1	M	1.32	1.01
CHAD		1.31	1.18
NCAM1		1.31	1.44
GSN		1.31	1.18
CDK14	M	1.30	1.43
KIAA1462	M	1.30	1.25
C3AR1		1.30	0.81
INHBA		1.29	1.09
P3H1		1.29	1.24
EDN1		1.29	0.66
TMPRSS2	E	1.28	1.11
TGFB2		1.28	0.96
PLEKHO1	M	1.28	0.74
PTEN		1.26	1.27
ITGB1BP1		1.26	1.24
SPARCL1	M	1.26	0.76
MPDZ	M	1.25	1.06
EPHA1		1.25	1.18
ANXA2P2		1.25	1.15
TPSB2		1.25	1.18
VEGFC		1.25	1.12
NFKB1		1.25	1.03
FBLN5		1.25	0.44
ETV4		1.25	0.91

Supplementary Table 1. A list of genes upregulated in PTX-treated tumors (fold change >1.25) compared to control tumors. 770 genes that contribute to cancer progression were quantitated, and their relative expression levels in PTX-treated tumors compared to vehicle-treated tumors were listed. RNA samples were prepared from a mixture of 10 tumors in 2 independent experiments.

Supplementary Table 2

Patient No.	Periostin score Mammotome	Neoadjuvant chemotherapy	Therapeutic effect	Periostin score surgical specimen
36	35.9	PTX + FEC	0	43.3
45	45.3	Nab-PTX + FEC	0	66.3
3	15.0	Nab-PTX + FEC	1a	59.7
7	48.3	FEC	1a	30.3
13	33.3	PTX + FEC	1a	50.0
28	41.3	Eribulin + FEC	1a	71.0
37	53.7	PTX + FEC	1a	56.3
39	38.7	PTX + FEC	1a	46.0
41	39.0	PTX + FEC	1a	39.2
42	50.0	PTX + FEC	1a	72.3
5	16.7	Nab-PTX + FEC	1b	64.3
10	34.7	Nab-PTX + FEC	1b	55.0
12	40.3	Nab-PTX	1b	76.0
14	26.0	PTX	1b	47.3
16	36.7	Nab-PTX + FEC	1b	45.1
19	51.0	Nab-PTX + FEC	1b	55.7
23	40.7	Eribulin + FEC	1b	75.0
8	33.7	PTX + FEC	2a	25.0
9	54.0	Nab-PTX + FEC	2a	49.0
15	38.0	Nab-PTX + FEC	2a	67.3
22	11.5	Nab-PTX + FEC	2a	38.3
29	30.3	Nab-PTX + FEC	2a	44.0
34	42.6	PTX + Bev	2a	59.0
35	45.7	FEC	2a	46.7
38	43.7	PTX + FEC	2	35.7
40	44.3	PTX + FEC	2	51.7

Supplementary Table 2. A summary of chemotherapy treatment information and periostin expression analysis by immunohistochemistry in 26 pairs of human TNBC clinical samples. PTX: Paclitaxel, Nab-PTX: Protein-bound Paclitaxel, AC: Doxorubicin + Cyclophosphamide, FEC: 5-Fluorouracil + Epirubicin + Cyclophosphamide, Bev: Bevacizumab. Therapeutic effect was assessed histologically; grade 0: almost no change in invasive cancer cells, grade 1a: mild changes in invasive cancer cells regardless of the area, or marked changes are seen in less than one-third of cancer cells, grade 1b: marked changes in one-thirds or more of invasive cancer cells, grade 2a: marked changes in two-thirds or more of invasive cancer cells, grade 2b: less than a few clusters of invasive cancer cells.

Supplementary Figure 1

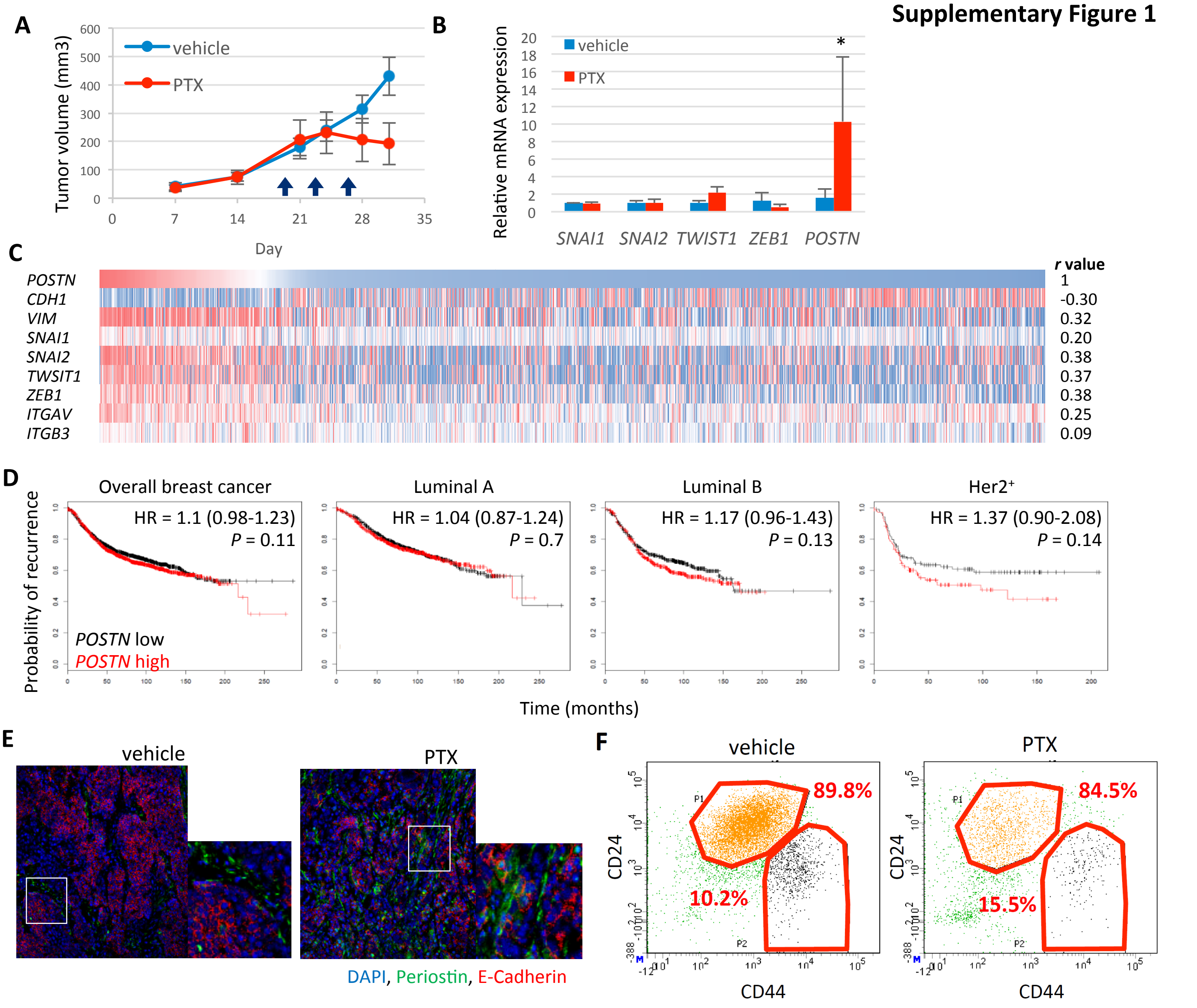

Supplementary Figure 1. POSTN expression correlates with an EMT gene signature and is upregulated by chemotherapy within an enriched CD44^{high}/CD24^{low} mesenchymal and tumor-initiating cell population in triple-negative breast cancer xenografts. **A**, Tumor growth curve in SUM149 xenografts upon PTX treatment. Arrows indicate times of drug administration. Tumor volume was measured on the indicated days and are presented as mean ± standard error (n = 6). **B**, Quantitative PCR analysis of indicated genes in tumor at the end of the experiment. Relative values are presented as mean ± standard error (n = 3). Relative values are presented as mean ± standard error, * $P < 0.02$, Student's *t* test. **C**, Correlation analysis of POSTN expression in 1,036 human cancer cell lines. Heatmap of gene expression and Pearson product-moment correlation coefficient (*r* value) are shown. *r* = .70 or higher: very strong correlation, *r* = .40 to .69: strong correlation, *r* = .30 to .39: moderate correlation, *r* = .20 to .29: weak correlation, *r* = .01 to .19: no or negligible correlation. **D**, Patient survival analysis in breast cancer patients (overall: n = 3554, Luminal A: n = 1764, Luminal B: n = 1002 and Her2+: n = 208) according to POSTN expression (low: 66.6%, high: 33.3%). Hazard ratio (HR), 95% confidence intervals. *P* value, log rank test. **E**, Immunostaining of periostin and E-cadherin in tumors collected at the end of the experiment. Right lower inserts are enlarged pictures of squared area. Scale bar, 100 µm. **F**, Flow cytometry analysis of tumors at the end of the experiment using CD44 and CD24 antibodies. Samples were prepared from a mixture of 6 tumors. Ratio of CD44^{high}/CD24^{low} to CD24high tumor cells is presented on the plots.

Supplementary Figure 2

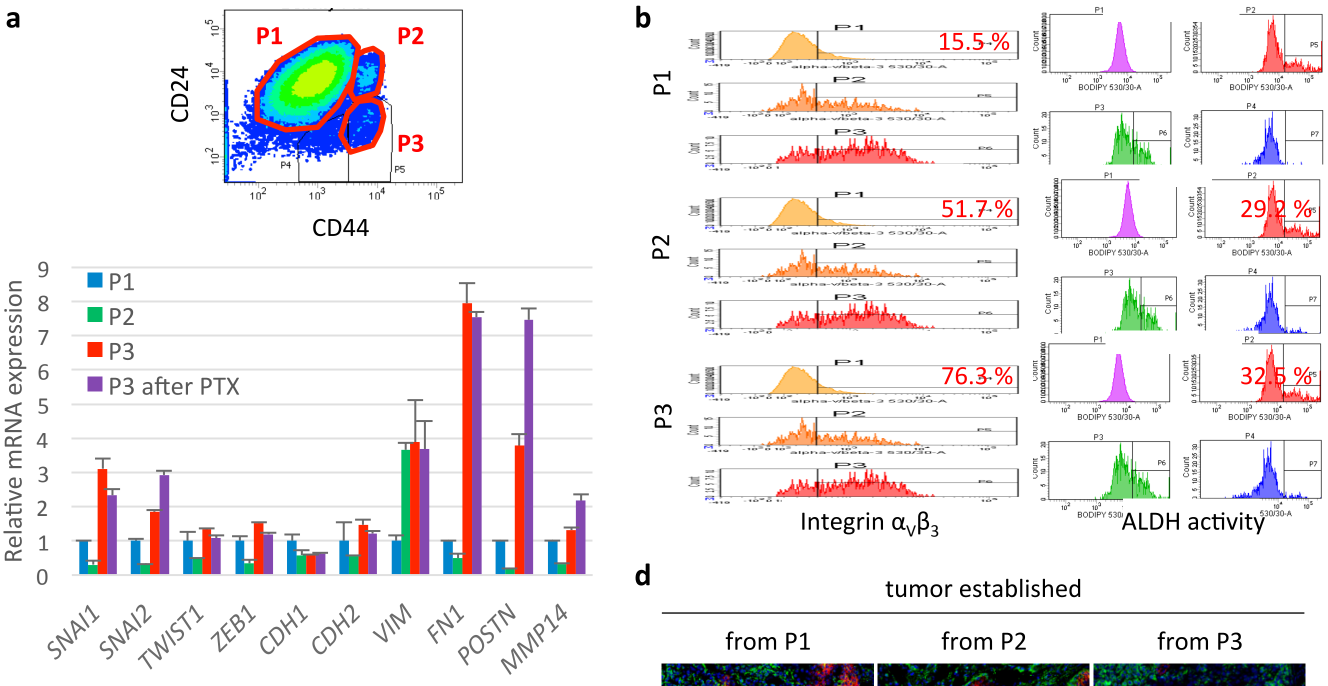

c

	1000 cells	100 cells	10 cells
P1	1/6 (16.7%)	0/6 (0%)	0/8 (0%)
P2	5/6 (83.3%)	2/6 (33.3%)	0/8 (0%)
P3	6/6 (100%)	4/6 (66.7%)	0/8 (0%)

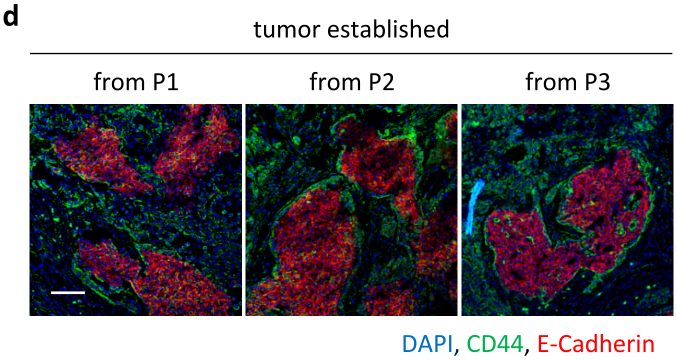

Supplementary Figure 2. The CD44^{high}/CD24^{low} mesenchymal population showed higher expression of periostin and enriched for tumor-initiating cells. **A**, Quantitative PCR analysis of indicated genes in the sorted CD44^{low}/CD24^{high} (P1), CD44^{high}/CD24^{high} (P2) and CD44^{high}/CD24^{low} (P3) populations. P3 population after PTX treatment was also analyzed. Relative values are presented as mean ± standard error. **B**, Flow cytometry analysis of MCF10DCIS tumor. P1, P2 and P3 populations were analyzed using Integrin αVβ3 antibody and ALDH enzymatic assay. Samples were prepared from the mixture of 10 tumors. Percentages of each population are presented on the plots. **C**, Tumorigenicity assay of sorted P1, P2 and P3 population in nude mice. After injection of the indicated numbers of cells, tumor formation was monitored for up to 3 months. **D**, Immunostaining of CD44 and E-Cadherin in tumors established from P1, P2 and P3 population. Scale bar, 100 µm.

Supplementary Figure 3

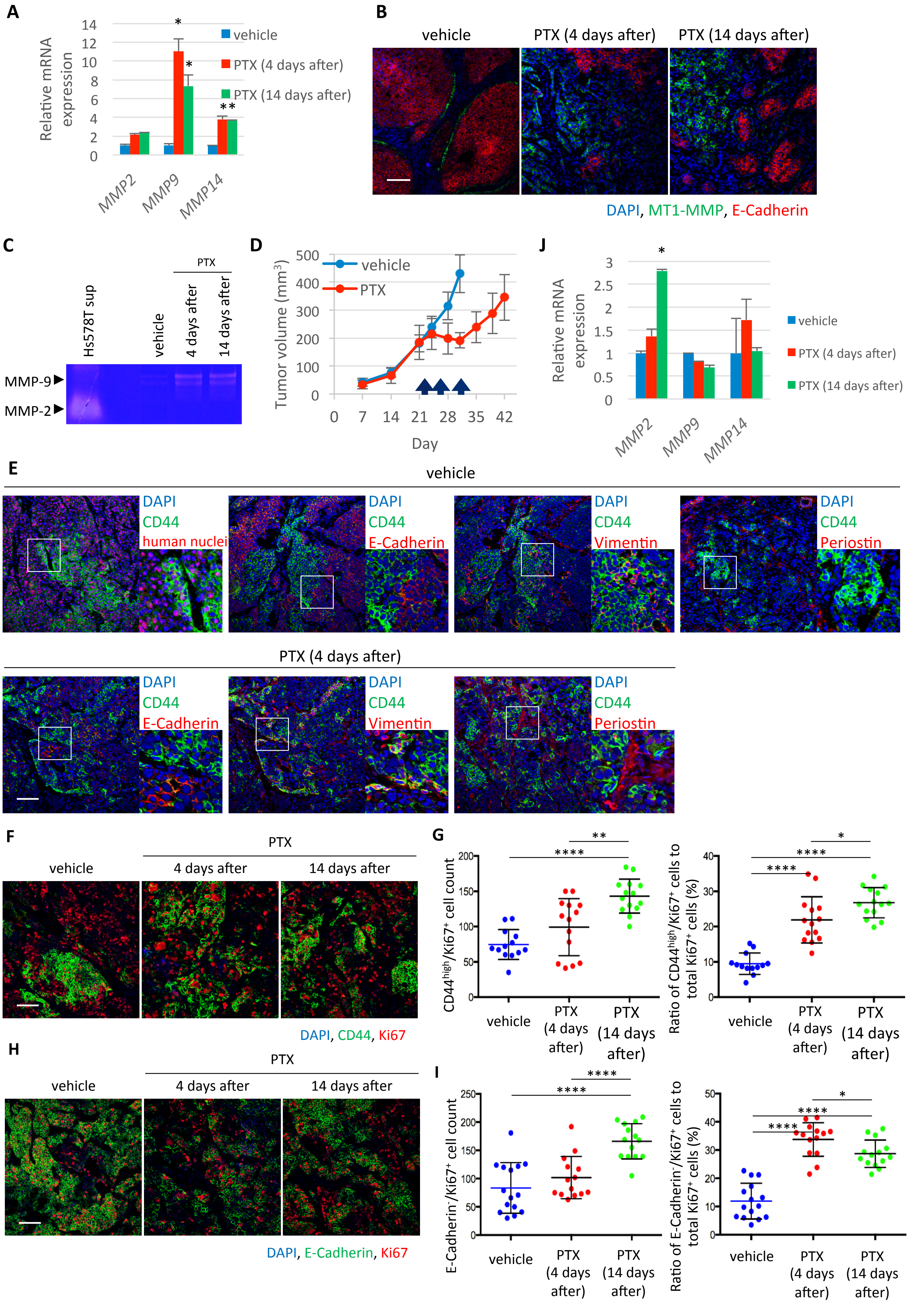

Supplementary Figure 3. Chemotherapy triggered mesenchymal cell expansion and tumor invasion in residual tumor. **A**, Quantitative PCR analysis of human MMP2, MMP9, and MMP14 in MCF10DCIS tumors. Samples were prepared from a mixture of 10 tumors. Relative values are presented as mean ± standard error. Relative values are presented as mean ± standard error, * $P < 0.02$, Student's *t* test. **B**, Immunostaining of periostin and MT1-MMP in MCF10DCIS tumors. Scale bar, 100 µm. **C**, Gelatin zymography analysis of tumor lysates. Samples were prepared from a mixture of 10 tumors. Culture supernatant of Hs578T breast cancer cells was used as a positive control. **D**, Tumor growth curve of SUM149 in response to PTX treatment. Arrows indicate times of drug administration. Tumor volume was measured on the indicated days. Data are presented as mean ± standard error (n = 6). **E**, Immunostaining of CD44, E-cadherin, vimentin and periostin in PTX-treated SUM149 tumors at Day 30. Right lower inserts are enlarged pictures of squared area. Scale bar, 100 µm. **F**, Immunostaining of CD44 and Ki67 in SUM149 tumors. Scale bar, 100 µm. **G**, The number of $CD44^{high}/Ki67^{+}$ SUM149 cancer cells per field were counted (left panel, n = 15), and the ratio of $CD44^{high}/Ki67^{+}$ cancer cells against total $Ki67^{+}$ cells was calculated (right panel, n = 15). Bars represent mean, and error bars represent standard deviation. * $P < 0.05$, ** $P < 0.01$, **** $P < 0.0001$, Mann-Whitney's test. **H**, Immunostaining of E-Cadherin and Ki67 in SUM149 tumors. Scale bar, 100 µm. **I**, The number of $E\text{-}Cadherin^{-}/Ki67^{+}$ SUM149 cancer cells per field were counted (left panel, n = 15), and the ratio of $E\text{-}Cadherin^{-}/Ki67^{+}$ cancer cells against total $Ki67^{+}$ cells was calculated (right panel, n = 15). Bars represent mean, and error bars represent standard deviation. * $P < 0.05$, **** $P < 0.0001$, Mann-Whitney's test. **J**, Quantitative PCR analysis of human MMP2, MMP9, and MMP14 in SUM149 tumors. Samples were prepared from a mixture of 6 tumors. Relative values are presented as mean ± standard error. Relative values are presented as mean ± standard error, * $P < 0.02$, Student's *t* test.

Supplementary Figure 4

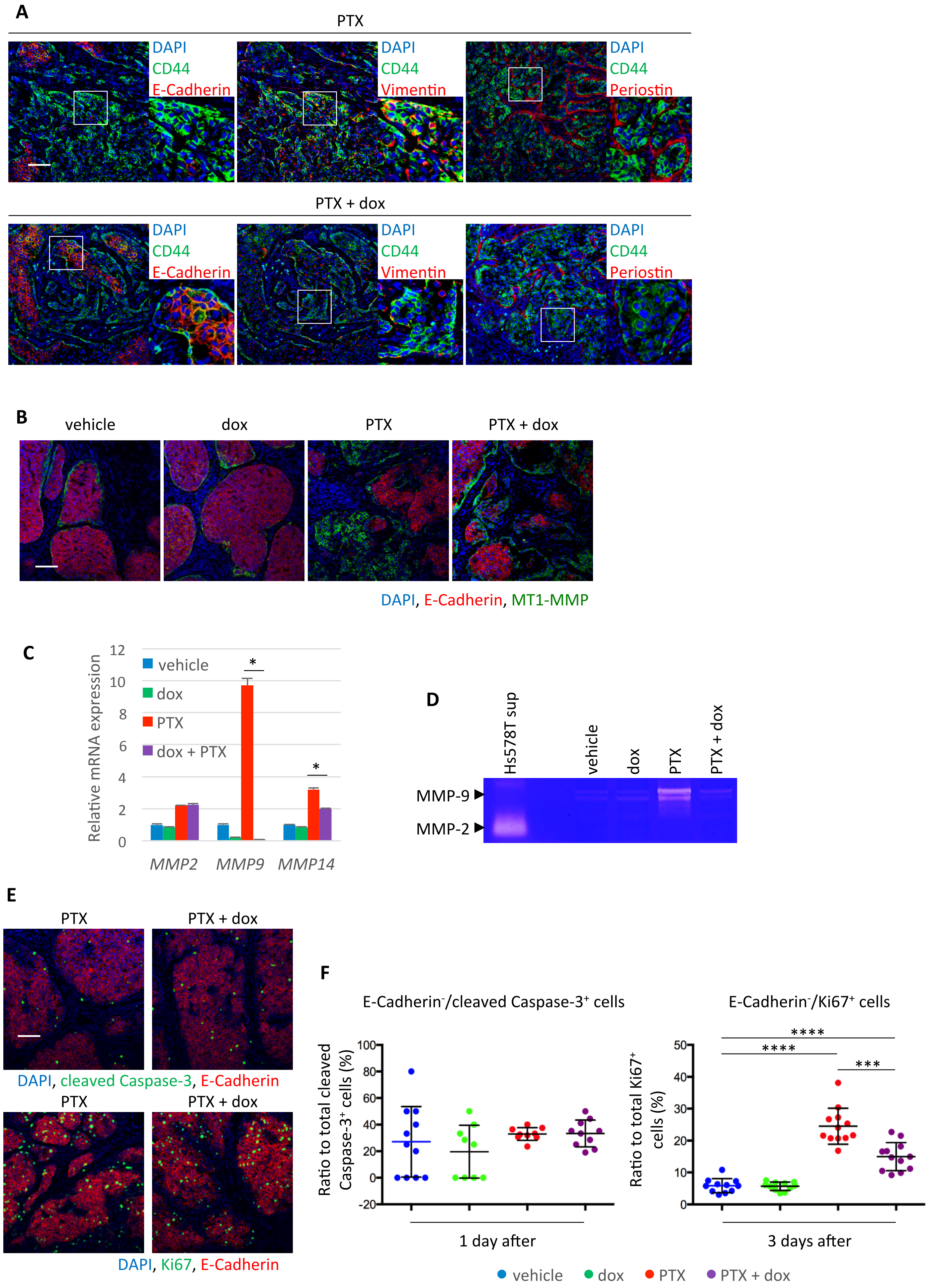

Supplementary Figure 4. Periostin knockdown inhibited mesenchymal cell expansion and tumor invasion after chemotherapy. **A**, Immunostaining of CD44, E-cadherin, vimentin and periostin in PTX-treated MCF10DCIS tumors collected at Day 30. Right lower inserts are enlarged pictures of squared area. Scale bar, 100 µm. **B**, Immunofluorescence staining of E-cadherin and MT1-MMP in tumors collected at Day 30. Scale bar, 100 µm. **C.** Quantitative PCR analysis of human MMPs in tumors collected at Day 30. Samples were prepared from a mixture of 10 tumors. Relative values are presented as mean ± standard error. Relative values are presented as mean ± standard error, * $P < 0.02$, Student's *t* test. **D**, Gelatin zymography analysis of tumor lysates at Day 30. Samples were prepared from a mixture of 10 tumors. Conditioned media from Hs578T cells was used as a positive control. **E**, Immunostaining of E-cadherin, cleaved caspase-3 and Ki67 in tumors. Tumors were sampled 1 or 3 days after one dose of PTX treatment. Scale bar, 100 µm. **F**, The number of E-cadherin$^-$/Ki67$^+$ cancer cells per field were counted (left panel, n = 10), and the ratio of E-cadherin$^-$/Ki67$^+$ cancer cells against total Ki67$^+$ cells was calculated (right panel, n = 10). Bars represent mean, and error bars represent standard deviation. *** $P < 0.001$, **** $P < 0.0001$, Mann-Whitney's test.

Supplementary Figure 5

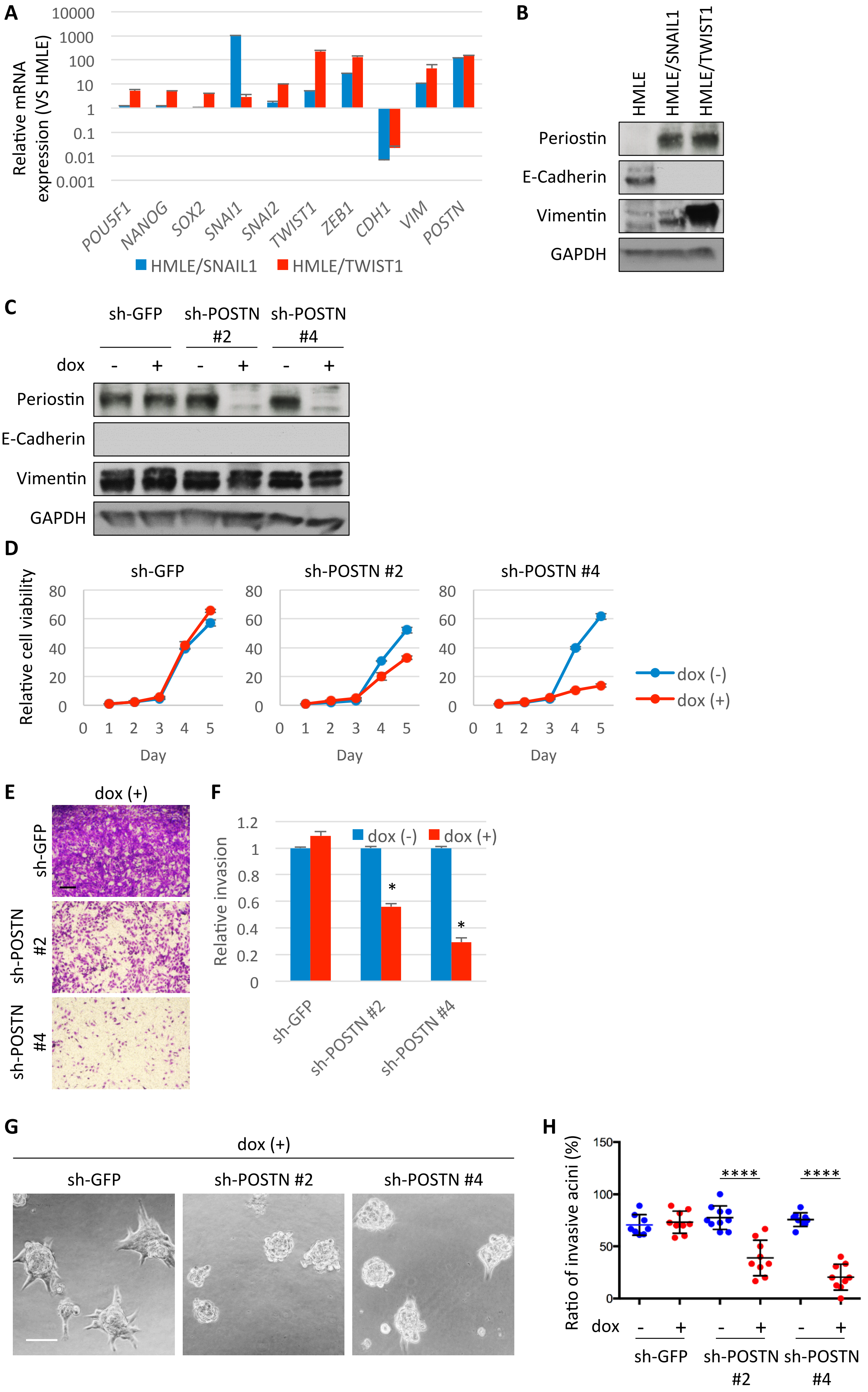

Supplementary Figure 5. Periostin is induced during TWIST1- or SNAIL1-induced EMT. **A**, Quantitative PCR analysis of indicated genes in SNAIL1 or TWIST1-transduced HMLE and parental HMLE cells. Relative values are presented as mean ± standard error from technical replicates. **B**, Western blot analysis of periostin, E-cadherin and vimentin in SNAIL1 or TWIST1-transduced HMLE and parental HMLE cells. **C**, Western blot analysis of periostin, E-cadherin and vimentin in HMLE/TWIST1 cells transduced with dox-inducible shRNAs as indicated. Cells were treated with doxycycline or control for 5 days. **D**, Cell growth analysis of HMLE/TWIST1 cells transduced with dox-inducible shRNAs as indicated. Cell numbers were measured on the indicated days. Relative values are presented as mean ± standard error (n = 3). **E**, Transwell invasion assay of HMLE/TWIST1 cells. Cells were preincubated with or without dox for 2 days. After 2 days culture on GFR-Matrigel-coated Transwell inserts, infiltrating cells were stained with crystal violet. Scale bar, 200 μm. **F**, Quantification of the transwell invasion assay in **E**. * $P < 0.05$, Student's *t* test. **G**, Organoid culture of HMLE/TWIST1 cells expressing indicated shRNAs. Cells were cultured for 6 days with or without dox in Matrigel. Scale bar, 100 μm. **H**, Ratio of invasive acini in organoid culture of HMLE/TWIST1 (n = 10). Cells were cultured for 6 days with or without dox in Matrigel. Bars represent mean, and error bars represent standard deviation. **** $P < 0.0001$, Mann-Whitney's test.

Supplementary Figure 6

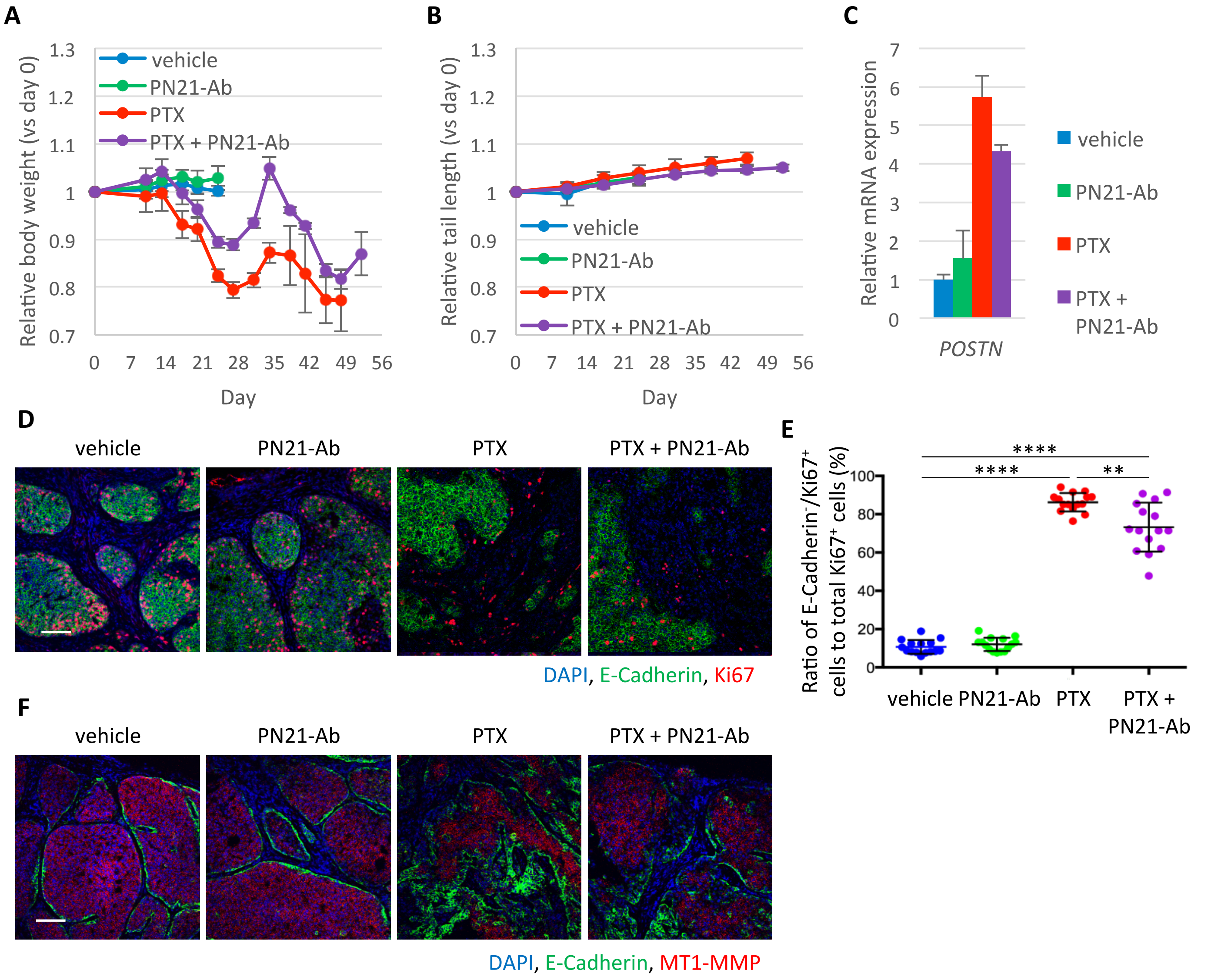

Supplementary Figure 6. An antibody targeting cancer-specific periostin variants inhibited mesenchymal cell expansion and tumor invasion after chemotherapy without any toxicity enhancement. **A**, Body weight curve of mice carrying MCF10DCIS tumors and treated with PTX and PN21-Ab as indicated. Body weight was measured on the indicated days. Data are presented as mean ± standard error (n = 5). **B**, Tail length curve of mice carrying MCF10DCIS tumors and treated with PTX and PN21-Ab as indicated. Tail length was measured on the indicated days. Data are presented as mean ± standard error (n = 5). **C**, Quantitative PCR analysis of indicated genes in tumors at Day 24. Samples were prepared from a mixture of 10 tumors. Relative values are presented as mean ± standard error from technical replicates. **D**, Immunostaining of E-cadherin and Ki67 in tumors collected at Day 24. Scale bar, 100 μm. **E**, The ratio of E-cadherin^{-}/Ki67^{+} cancer cells against total Ki67^{+} cells was calculated in tumors collected at Day 24 (n = 15). Bars represent mean, and error bars represent standard deviation. ** $P < 0.01$, **** $P < 0.0001$, Mann-Whitney's test. **F**, Immunostaining of E-cadherin and MT1-MMP in tumors collected at Day 24. Scale bar, 100 μm.
